# Primary care use among adults with eating disorders in England: a population-based cohort study using electronic health records

**DOI:** 10.1136/bmjopen-2026-119143

**Published:** 2026-06-24

**Authors:** Jessica Wilkins, Lucy Gallagher, Karina L Allen, Alexandru Dregan, Chloe Gao, Jamie Scuffell, Ulrike Schmidt

**Affiliations:** 1 Centre for Research in Eating and Weight Disorders, Institute of Psychiatry, Psychology and Neuroscience, King’s College London, London, UK; 2 Eating Disorders Outpatients Service, South London and Maudsley NHS Foundation Trust, London, UK; 3 Department of Psychological Medicine, Institute of Psychiatry, Psychology and Neuroscience, King’s College London, London, UK; 4 Faculty of Medicine, The University of British Columbia, Vancouver, British Columbia, Canada; 5 Department of Population Health Sciences, School of Life Course and Population Sciences, King’s College London, London, UK

**Keywords:** Primary Care, Health Equity, MENTAL HEALTH, Eating disorders, Electronic Health Records

## Abstract

**Abstract:**

**Objectives:**

To examine primary care contacts among individuals with eating disorders (EDs) and assess differences across diagnoses and ethnic backgrounds.

**Design:**

Matched cohort study using retrospective primary care data.

**Setting:**

Primary care electronic health records from the Clinical Practice Research Datalink (CPRD) and linked Hospital Episode Statistics (HES) covering 1 January 2010 to 31 December 2023.

**Participants:**

46 473 individuals aged 18–65 years, with a recorded ED diagnosis or a referral to ED services, matched by age, sex and practice location (ratio 1:3) to 145 286 individuals without an ED.

**Primary/secondary outcome measures:**

The primary outcome was the number of primary care contacts in 24 months prior to ED diagnosis or referral to ED specialist service. Secondary outcomes examined whether ethnicity impacted likelihood of referral to specialist ED services.

**Results:**

Most individuals had a diagnosis of anorexia nervosa (43.0%), followed by bulimia nervosa (13.8%) and other specified feeding and ED (6.6%). 40.6% were aged 18–25 years, 79.5% were female and ethnicity was predominantly White (83.7%), with smaller proportions Asian (6.3%) and Black (3.4%). Compared with non-ED controls, cases had approximately double the rate of primary care contacts (incidence rate ratio (IRR)=1.96, 95% CI 1.94 to 1.98). Elevated contact rates were observed across all ED diagnostic groups, with IRRs ranging from 1.78 (95% CI 1.75 to 1.82) for anorexia nervosa to 2.45 (95% CI 2.09 to 2.88) for avoidant/restrictive food intake disorder (all p<0.001). Contact rates were significantly lower across all minoritised ethnic groups compared with White individuals. Referral odds were significantly lower among Asian (OR=0.78, 95% CI 0.65 to 0.94) and Black patients (OR=0.56, 95% CI 0.41 to 0.79).

**Conclusions:**

There is a need to increase ED awareness within minoritised communities, alongside culturally inclusive primary care adaptations to support help-seeking for EDs. Targeted education for clinicians and patients in primary care may also improve screening and recognition of EDs across diverse presentations and communities, facilitating access to timely, evidence-based care.

**Data availability statement:**

The dataset from this study is held securely at the Medicines and Healthcare products Regulatory Agency. Access may be granted on completing a data request. The CPRD Ethnicity Record sources underlying data from HES and primary care data copyright 2025, re-used with the permission of The Health & Social Care Information Centre.

**STRENGTHS AND LIMITATIONS OF THIS STUDY**

- This study examined primary care contacts among individuals with eating disorders (EDs), using a large, nationally representative electronic health record (EHR) dataset.
- Routinely collected EHR data enabled detailed examination of differences in service use by ethnicity and diagnosis, extending beyond treatment-based cohorts or self-reported community samples.
- ED diagnoses and primary care contacts rely on routine clinician coding practices, which may vary and lead to misclassification or incomplete recording.
- The dataset excludes individuals who are not registered with, or do not access primary care.
- Use of broadly defined ethnic categories may obscure important within-group differences in primary care use among individuals with EDs.

## Introduction

 Eating disorders (EDs), including anorexia nervosa (AN), bulimia nervosa (BN) and binge eating disorder (BED), are serious mental illnesses associated with substantial psychological and physical morbidity and elevated mortality rates.^1^ However, despite their significant negative impact on quality of life, most individuals with EDs do not seek treatment.^2 3^ Although EDs are known to occur across all demographic groups (age, gender, ethnicity), individuals from minoritised ethnic backgrounds are under-represented in clinical populations and in research studies.^4^ Understanding how individuals access ED care is therefore critical, particularly given substantial regional and demographic differences in referral and treatment uptake.^2^

Primary care is frequently the first point of contact for individuals with EDs, with some studies reporting that up to 50% of cases were first identified during a primary care visit.^3 5 6^ Being asked about mental health in primary care increases the likelihood of receiving mental health treatment, demonstrating that such conversations may facilitate uptake of specialist care.^7 8^ Additionally, healthcare use is elevated among individuals with EDs, highlighting the role of primary care in the detection and management of EDs.^5^,^7^ In the UK, most specialist ED services can only be accessed through General Practitioner (GP) referral, making an understanding of primary care contacts for individuals with EDs particularly important.

EDs affect individuals from minoritised ethnic backgrounds at rates comparable to those observed among White individuals with more commonalities than differences in underlying risk factors across ethnic groups.^9^ However, evidence from the UK, USA and Europe demonstrates that individuals from minoritised ethnic backgrounds are less likely to be referred for ED treatment.^4 10 11^ A systematic review of access to secondary mental healthcare services in the UK revealed that, compared with White patients, Black patients were less likely to be referred to specialist services via primary care and more likely to access these services by complex routes including via the justice system.^12^ These findings were confirmed in a sample of young people accessing general mental health services in South East London in 2021.^13^ Research highlights unique barriers for people from minoritised ethnic groups, including stigma around mental health and persistent stereotypes about who develops EDs, alongside facilitators such as improved cultural competence among clinicians and greater representation within services.^14^ It also identifies the important role of primary care in recognising symptoms and facilitating referral to specialist support.^4^ Taken together, this highlights the central role of primary care interactions in shaping pathways into specialist treatment and suggests that differential experiences within primary care may contribute to observed inequities in access to ED services.

The first study to use Clinical Practice Research Datalink (CPRD) data to examine ED presentations in primary care was conducted approximately 30 years ago and found higher rates of primary care consultations among ED patients relative to those without EDs.^15^ A more recent study found only a small proportion of adolescents consulting with EDs in primary care were referred to specialist services.^2^ These studies focused predominantly on the experiences of individuals living with AN and BN and less on other EDs including BED, other specified feeding or eating disorder (OSFED), and avoidant/restrictive food intake disorder (ARFID).

To our knowledge, no previous studies have compared primary care consultation patterns among individuals with different EDs or examined how demographic factors, such as ethnicity, influence inequalities in care utilisation and referral to specialist services.

### Study aims

The overall objective of this study was to understand patterns in primary care contacts for people diagnosed with EDs in England and Northern Ireland. Our research questions were:

1. Do primary care contact rates during the 24 months preceding an ED diagnosis/referral differ between individuals with EDs compared with non-ED controls, matched by age, sex and practice location?
2. Among individuals with EDs, to what extent do contact rates during the 24 months preceding diagnosis or referral vary by ethnic background?
3. Among individuals with EDs, does ethnicity predict differences in referral patterns for specialist ED treatment?

## Methods

Ethical approval for this study was granted by the CPRD’s Research Data Governance Process (protocol reference number 24_004014). All patient data are anonymised and thus the requirement for patient consent was waived. Patients can opt-out of sharing their records with CPRD even if their practice routinely provides information to the database. This study was reported in accordance with the Strengthening the Reporting of Observational Studies in Epidemiology (STROBE) and The Reporting of studies Conducted using Observational Routinely-collected health Data (RECORD) reporting guidelines (SM 1). We worked with an advisory group of young people with lived experience of EDs (aged 16–25 years) who offered feedback on the protocol of this study.

### Study design

A matched cohort observational study was conducted using the UK CPRD database.

### Data sources

CPRD is an electronic health record (EHR) database comprising primary care data from GP practices across the UK. It includes anonymised data for over 65 million historical patients and is broadly representative across ethnicity, gender and age compared with the UK population.^16^,^19^ At the time of writing, approximately 30% of GP practices (n=~2400) in the UK contributed data to CPRD, comprising around 30% (19 million) of the UK population.^19 20^ CPRD contains detailed primary care data including medical diagnoses, prescriptions, demographics, clinical events, lab tests and referrals. Furthermore, CPRD data was linked to Hospital Episode Statistics (HES) data to complement primary care data on ethnicity.

### Study population

Data for the present study were obtained from the CPRD Aurum database, which comprises GP practices in England and Northern Ireland (small number of practices) only, and the observation period extended from 1 January 2010 to 31 December 2023. Eligible individuals were adults aged 18–65 years who had at least 24 months of continuous GP registration. This age range was consistent with comparable CPRD studies examining EDs and mental health populations, facilitating comparison with existing literature.^21 22^ Healthcare use was observed over a 24-month period. This window was chosen to capture the prolonged period of untreated illness commonly observed prior to ED diagnosis and referral.^23^ Cases were identified either by a diagnosis of an ED, identified using Read and/or Systematised Nomenclature of Medicine Clinical Terms (SNOMED CT) medical codes corresponding to ED diagnoses or by a referral to specialist ED services.

The index date was defined as the first date of ED diagnosis during the follow-up period, or the date of referral to an ED service where no diagnosis was recorded. Individuals referred to specialist ED services without a recorded ED diagnosis in primary care were included to capture probable ED presentations where GPs may not have assigned an ED diagnostic code prior to referral. GP records were extracted for the 24 continuous months prior to this index date. Each patient with an ED was exactly matched to up to three controls without an ED on age, sex, GP practice ID and index date. Controls were required to have no recorded ED diagnosis or referral in their CPRD record, based on the study codelists (table 1). They were assigned the same index date as their matched case. To ensure this was a valid time point for the control, we required the cases’ index date fell within the control’s period of active registration at the same practice. This ensured that the controls were under clinical observation during the same time as their matched case, reducing the likelihood of selecting controls with infrequent primary care use. It also helped to account for temporal changes in clinical practice, coding behaviour and EHR recording over the study period.

**Table 1:** Available data and measures

Data item	Data source/codelist	Description
ED Diagnosis	CPRD Aurum (SM 2.1)	Previously validated ED codelists were updated for the present study to identify Read and/or SNOMED CT medical codes corresponding to categories from DSM-IV, DSM-5 and ICD-10. Only codes representing clinical ED diagnoses were included; non-specific symptom codes (eg, loss of appetite) were excluded. The terms ‘appetite loss—anorexia’ and ‘anorexia’ were retained, as excluding them may have missed records where this shorthand was used for AN in primary care coding. EDs were categorised as AN, BN, BED, ARFID and OSFED. Atypical AN and atypical BN were grouped with AN and BN respectively to reflect evidence that atypical and typical presentations do not differ in clinical impairment.^54^
Ethnicity	CPRD and HES	Ethnicity categories are defined by Office of National Statistics codes: White, Black, Asian, Mixed ethnic background, Other ethnic group. Where individuals have more than one ethnicity recorded, the more recent record is prioritised. HES data complements ethnicity data in primary care, increasing completeness to around 88% (from 67%).^20^ Evaluation of the ethnicity record demonstrates that CPRD data is representative of the English population compared with results from the UK census.^16^ This study included person-level linkage between CPRD Aurum primary care records and linked HES data for ethnicity. Linkage is performed by CPRD using the CPRD ethnicity algorithm and only records meeting CPRD linkage quality standards are made available for research use.
BMI	CPRD Aurum (SM 2.2)	Previously validated codelists using weight, height and BMI were revised for use with an adult cohort. Completeness of BMI in CPRD is increasing over time and women are more likely to have BMI recorded in CPRD compared with men.^18^ BMI was categorised according to WHO guidelines as underweight (<18.5 kg/m²), normal weight (18.5–24.9 kg/m²), overweight (25.0–29.9 kg/m²), obesity class I (30.0–34.9 kg/m²) and obesity class II or higher (≥35.0 kg/m²).
IMD quintile	Linked data provided by CPRD via ONS	Ranks geographical areas according to deprivation across seven domains including income, employment, health, education, crime, housing and the living environment.^55^ Quintiles range from the first (most deprived 20%) to the fifth (least deprived 20%). Completeness of these socioeconomic status data in CPRD is high relative to the broader UK population, with evidence of comparatively greater representation from more deprived, urban-area practices.^17^ This study included practice-level linkage to ONS deprivation data. Linkage is performed by CPRD using established NHS Digital methodologies and only records meeting CPRD linkage quality standards are made available for research use.
Smoking status	CPRD Aurum	Defined using previously validated smoking status codelists developed for use in CPRD.^56^ Participants were categorised as ever having smoked (including both current and ex-smokers) and never having smoked.
Referral events	CPRD Aurum (SM 2.4)	A new codelist was developed by searching the CPRD database for observation medical codes (medcodeid) containing keywords commonly used to denote specialist ED services (eg, ‘referral to eating disorders clinic’, ‘referral to eating disorders outreach clinic’). Referral events were identified by linking recorded referral codes (medcodeid) to a predefined referral codelist. To improve validity, we restricted analyses to referral records that also contained a referral source and referral type, indicating that a referral had been initiated.

AN, anorexia nervosa; ARFID, avoidant restrictive food intake disorder; BED, binge eating disorder; BMI, body mass index; BN, bulimia nervosa; CPRD, Clinical Practice Research Database; DSM-5, Diagnostic and Statistical Manual of Mental Disorders fifth Edition; DSM-IV, Diagnostic and Statistical Manual of Mental Disorders fourth Edition; ED, eating disorder; HES, Hospital Episode Statistics; ICD, International Classification of Diseases; IMD, Index of Multiple Deprivation; ONS, Office of National Statistics; OSFED, other specified feeding and eating disorder; SNOMED CT, Systematised Nomenclature of Medicine Clinical Terms.

### Variables

Clinical events such as diagnoses, referrals, lab tests and treatment consultations are recorded in CPRD using standardised clinical coding systems, primarily SNOMED CT and Read medical codes. As multiple codes can represent the same clinical concept, curated codelists were used to group equivalent codes and ensure consistent identification of exposures and outcomes (eg, EDs, smoking status). We used previously published codelists alongside novel codelists developed for this study, following published guidance for routinely collected EHR data. All codelists were reviewed by clinicians with expertise in primary care and EDs.^24^ Full details of all codelists and checklists are provided in the online supplemental materials (SM 2.1–SM 2.5).

Table 1 summarises the data items included in the analyses, indicating the data source for each variable and the associated codelist where applicable.

### Consultation rates

We followed the approach detailed by Wyatt *et al*’s^25^ to identify primary care consultations. Full details of our consultation source codelists, job category lists and medcodeid lists can be found in online supplemental materials (SM 2.5). We derived the number of primary care contacts in the 24 months prior to ED diagnosis or referral to specialist service by two types of contact: face-to-face and telephone/virtual with any clinical staff. Telephone/virtual contacts were included because evidence demonstrates that these types of contacts have increased substantially in the years since the COVID-19 pandemic.^25^

Using consultation source codes, we first excluded codes that were administrative in nature (eg, ‘report’, ‘document’) or clearly outside the scope of primary care (eg, ‘Accident and Emergency’). Remaining codes were categorised either as ‘face-to-face’ or ‘telephone/virtual’ interactions. Consultation codes were then linked to clinical job categories and records associated with non-clinical staff (eg, ‘Receptionist’) were removed. We restricted the job code list to clinical staff roles to capture patient-clinician encounters rather than administrative activity. This approach is consistent with previous CPRD consultation-rate studies, for example,^22 25 26^ and reduced the risk of capturing administrative record entries or documentation of consultations rather than the consultation itself. Ambiguous consultation codes (eg, ‘Booked Appointment’) were required to have an associated observation (diagnostic) code from our list of consultation codes (SM 2.5). To reduce duplication, identical events recorded on the same date were removed,^22^ and consultation records from the first 90 days after the index date were excluded to minimise the impact of bulk-imported data following practice registration. We allowed a maximum of one consultation per patient per day and used clustered standard errors by practice or matched set to account for systematic coding differences across practices.

### Statistical methods

Data linkage and data preparation within CPRD Aurum was conducted using Stata (V.18), after which data were imported into R (V.2023.12.1+402) for all statistical analyses. Prior to analysis, CPRD data were cleaned and recoded into analytical variables, including identification of diagnosis, ethnicity, body mass index (BMI), smoking and deprivation categories. Primary care consultation counts were analysed using fixed-effects negative binomial regression to account for overdispersion in consultation counts and the matched case-control study design. To address Research Question 1, fixed-effects negative binomial regression models were used to compare consultation rates between individuals with EDs and non-ED controls, and to examine variation by diagnosis. Models compared individuals within matched case-control sets. Age, sex, GP practice and index date were therefore controlled by design through matching and by inclusion of matched-set fixed effects. Smoking status was included as a covariate because it was not part of the matching strategy and may be associated with healthcare use.^27^ Robust standard errors clustered at the matched-set level. To examine whether BMI accounted for part of the observed association, we re-estimated models with and without BMI adjustment. Results were materially unchanged for most diagnoses, and therefore models without BMI are presented as the primary analyses, with BMI-adjusted estimates provided in the online supplemental (SM 4.2). To address Research Question 2, we fitted a second fixed-effects negative binomial model to examine associations between ethnicity and consultation rates within matched case-control sets. Smoking status was included as a covariate, and White ethnicity was specified as the reference category because it was the largest group. All outcomes were ascertained within a fixed 24-month pre-index window for every participant (24 months prior to diagnosis/referral). As follow-up time was identical for all individuals, no offset for person-time was required and model estimates compare consultation counts over equivalent 24-month observation windows.

To address Research Question 3, whether ethnicity predicted a referral to ED specialist services, we fitted a multivariable logistic regression model with ever receiving an ED referral (yes/no) as the outcome. Cases included in the cohort on the basis of an ED referral alone, with no recorded ED diagnosis, were excluded from this analysis because referral was the outcome being modelled. An interaction between ethnicity and BMI category was included to assess potential effect modification, and models adjusted for age, sex, ED diagnosis, Index of Multiple Deprivation (IMD) quintile and smoking status. Results are reported as ORs with 95% CIs. In line with Bauer’s quantitative intersectionality framework, marginal effects and predicted probabilities were estimated for ethnicity-BMI combinations, with pairwise comparisons conducted using Tukey-adjusted tests.^28^ Additionally, given evidence that referral patterns and access to mental health services vary by geographic region in the UK^29^ a separate multivariable logistic regression model was fitted to examine whether referral to ED specialist services differed by region, adjusting for age, sex, ED diagnosis, IMD quintile and smoking status and the results are reported in SM 5.4.

Complete-case analysis was conducted for all primary models, whereby individuals with missing data on variables included in each model were excluded. To assess the robustness of findings to missing smoking data, we conducted pre-specified sensitivity analyses for Research Question 1 by (1) restricting analyses to individuals with complete smoking data and (2) re-running models including the full cohort with a separate category for missing smoking status. These sensitivity analyses did not materially change the results (SM 4.1). The proportion of missing data for key variables is reported in online supplemental material SM3 and results of sensitivity analyses are presented in SM4.1–SM4.2. We had originally planned to include hypokalaemia as a covariate as a proxy indicator of ED severity (SM 2.3), given its association with recurrent purging behaviours (eg, vomiting, laxative and diuretic abuse) and its occurrence in severe ED presentations.^30^ However, only a small proportion of participants met criteria for hypokalaemia (6.97%), and the variable was therefore excluded from the final analytical models.

## Results

### Patient characteristics

The cohort data contained a total of 46 473 individuals who had been diagnosed with an ED or referred to an ED service between 2010 and 2023. Most individuals (16 690; 43.0%) had a diagnosis of AN and the next most common diagnoses were BN (5370, 13.8%) and OSFED (2579, 6.6%). A substantial proportion of individuals had an ‘unspecified’ ED diagnostic label (12 581, 27.1%) and an additional 7682 (16.5%) did not have an ED diagnosis coded, meaning that they were referred to a specialist ED service without receiving a specific diagnosis in primary care. The largest proportion of cases were between 18 and 25 years of age (40.6%), followed by 26—35 year olds (23.1%) and most individuals were female (79.5%). Participant characteristics are detailed in table 2.

**Table 2:** Demographic and clinical characteristics of cases and matched controls

Characteristic	Cases (n=46 473)	Controls (n=1 45 286)	SMD
Sex—n (%)			0.007
Female	36 923 (79.5%)	115 817 (79.7%)	
Male	9550 (20.5%)	29 469 (20.3%)	
Ethnicity—n (%)			0.203
White	38 906 (83.7%)	110 452 (76.0%)	
Asian	2943 (6.3%)	14 291 (9.8%)	
Black	1597 (3.4%)	6111 (4.2%)	
Mixed	919 (2.0%)	3012 (2.1%)	
Unknown	1946 (4.2%)	10 055 (6.9%)	
Age—n (%)			0.015
18–25	18 878 (40.6%)	59 669 (41.1%)	
26–35	10 738 (23.1%)	33 766 (23.2%)	
36–45	6784 (14.6%)	20 752 (14.3%)	
46–55	5471 (11.8%)	16 577 (11.4%)	
56–65	4602 (9.9%)	13 406 (9.2%)	
IMD quintile—n (%)			0.008
IMD 1	7026 (15.1%)	22 248 (15.3%)	
IMD 2	6878 (14.8%)	21 631 (14.9%)	
IMD 3	8916 (19.2%)	27 977 (19.3%)	
IMD 4	11 796 (25.4%)	36 570 (25.2%)	
IMD 5	11 796 (25.4%)	36 570 (25.2%)	
ED diagnosis			
AN	16 690 (43.0%)	--	
ED-unspecified	12 581 (27.1%)	--	
BN	5370 (13.8%)	--	
OSFED	2579 (6.6%)	--	
BED	731 (1.9%)	--	
Pica	522 (1.3%)	--	
ARFID	318 (0.8%)	--	
Mixed ED	4271 (9.2%)	--	
No ED diagnosis (referral only)	7682 (16.5%)	--	
BMI			0.420
<18.5 (underweight)	6253 (15.9%)	4674 (4.7%)	
18.5–24.9 (normal)	16 200 (41.3%)	41 520 (42%)	
25–29.9 (overweight)	7193 (18.3%)	26 617 (26.9%)	
30–34.9 (Obese I)	4245 (10.8%)	14 505 (14.7%)	
>35 (Obese II)	5324 (13.6%)	11 481 (11.6%)	

ED diagnosis was defined using the diagnosis closest to the index date (start date of data extraction). Individuals with multiple ED diagnoses were classified as mixed eating disorder which is why numbers exceed 46 473. ED-unspecified includes diagnoses without further specification (see online supplemental material for codelist). BMI categories were defined using WHO criteria based on the most recent pre-index BMI. Percentages are calculated within case and control groups. Standardised mean differences (SMDs) were used to assess balance between cases and controls. Values<0.1 indicate good balance.^57^

AN, anorexia nervosa; ARFID, avoidant/restrictive food intake disorder; BED, binge eating disorder; BMI, body mass index; BN, bulimia nervosa; ED, eating disorder; IMD, Index of Multiple Deprivation; OSFED, other specified feeding or eating disorder; SMD, standardised mean difference.

### Research Question 1: Do primary care contact rates during the 24 months preceding an ED diagnosis/referral differ between individuals with EDs compared to non-ED controls, matched by age, sex and practice location?

Individuals with an ED had a higher mean number of total primary care contacts than controls over the 2 years prior to the index date (33.1 vs 20.2), including face-to-face contacts (14.9 vs 10.1) and telephone or virtual contacts (14.3 vs 7.4). Furthermore, contact rates were markedly higher among female cases compared with male cases (34.3 vs 28.3). In fixed-effects negative binomial regression analyses, adjusted for matched-set factors (age, sex, practice and index date) and smoking status, cases had almost twice the rate of primary care consultations compared with controls (IRR=1.96, 95% CI 1.94 to 1.98).

Contact rates differed substantially across ED diagnoses (reference=non ED controls). All ED diagnostic groups had significantly higher contact rates than controls, with incidence rate ratios (IRRs) ranging from 1.78 for AN (95% CI 1.75 to 1.82) to 2.45 for ARFID (95% CI 2.09 to 2.88). Elevated rates were also observed for all other diagnoses (table 3). Adjustment for BMI in sensitivity analyses resulted in only modestly different diagnosis-specific IRRs, indicating that differences in contact rates were not primarily explained by BMI, except for BED where the IRR decreased by approximately 15% after BMI adjustment (online supplemental materials 4.2). The non-adjusted model included 161 698 individuals; the BMI-adjusted sensitivity model included 123 204.

**Table 3:** Association between eating disorder (ED) diagnosis and number of primary care contacts in a matched case-control cohort (n=1 61 698)

Diagnosis	IRR	95% CI	P value
Non-ED controls	1.00	Reference	–
AN	1.78	1.75–1.82	<0.001
BN	1.88	1.83–1.93	<0.001
OSFED	1.83	1.76–1.90	<0.001
BED	2.08	1.91–2.27	<0.001
ARFID	2.45	2.09–2.88	<0.001
Pica	2.25	2.07–2.45	<0.001
ED-unspecified	2.04	2.00–2.08	<0.001
Mixed ED	2.32	2.24–2.41	<0.001

Incidence rate ratios and 95% CIs were estimated using fixed-effects negative binomial regression models. Models compared individuals within matched case-control sets and therefore controlled for age, sex, GP practice and index date through matched-set fixed effects. Models were additionally adjusted for smoking status. Non-ED controls were used as the reference group.

AN, anorexia nervosa; ARFID, avoidant/restrictive food intake disorder; BED, binge eating disorder; BN, bulimia nervosa; ED, eating disorder; ED-unspecified, unspecified eating disorder; GP, general practitioner; IRR, incidence rate ratio; OSFED, other specified feeding and eating disorder.

### Research Question 2: Among individuals with EDs, to what extent do contact rates during the 24 months preceding diagnosis or referral vary by ethnic background?

Consultation rates were significantly lower among Asian, Black, Mixed, Other and Unknown ethnicity groups compared with White participants (table 4).

**Table 4:** Association between ethnicity and number of primary care consultations in a matched case-control cohort (n=1 61 372)

Variable	Category	IRR	95% CI	P value
Ethnicity	White	1.00	Reference	–
	Asian	0.82	0.80–0.85	<0.001
	Black	0.84	0.81–0.88	<0.001
	Mixed	0.86	0.82–0.91	<0.001
	Other	0.48	0.44–0.53	<0.001
	Unknown	0.75	0.73–0.78	<0.001

Incidence rate ratios and 95% CIs were estimated using fixed-effects negative binomial regression models. Models compared individuals within matched case-control sets and therefore controlled for age, sex, GP practice and index date through matched-set fixed effects. Models were additionally adjusted for smoking status. White ethnicity and never smoker were used as the reference categories.

GP, general practitioner; IRR, incidence rate ratio.

This model was repeated with an interaction term between ethnicity and ED case status to assess whether the impact of having an ED on primary care consultation rates differed across ethnic groups. The relative increase in consultation rates associated with ED case status varied by ethnicity (table 5). Compared with White individuals, the relative increase in consultation rates between ED cases and controls was significantly greater among Asian (IRR 1.12), Black (IRR 1.24), Mixed (IRR 1.12) and Other ethnic groups (IRR 1.64).

**Table 5:** Association between ED case status and primary care consultation rates, stratified by ethnicity (n=1 61 372)

Ethnicity	Cases vs controls IRR	95% CI	P value
White (ref)	1.90	1.88–1.93	<0.001
Asian	2.14	2.03–2.25	<0.001
Black	2.36	2.21–2.53	<0.001
Mixed	2.14	1.93–2.36	<0.001
Other	3.12	2.56–3.80	<0.001
Unknown	1.90	1.79–2.02	<0.001

Incidence rate ratios and 95% CIs were estimated using fixed-effects negative binomial regression models including an interaction between ethnicity and ED case status. Models compared individuals within matched case-control sets, therefore controlling for age, sex, GP practice and index date through matched-set fixed effects. Models were additionally adjusted for smoking status. White ethnicity and non-ED controls were specified as the reference categories. IRRs compare primary care consultation rates between cases and controls within each ethnic group.

ED, eating disorder; GP, general practitioner; IRR, incidence rate ratio.

### Research Question 3: Among individuals with EDs, does ethnicity predict differences in referral patterns for specialist ED treatment?

Overall, the proportion of cases who received a referral to specialist ED services was low (23.9%; n=11 120). After excluding individuals with no recorded ED diagnosis and restricting to complete cases, the adjusted analysis included 32 187 participants. Ethnicity was associated with the likelihood of receiving an ED referral. At normal BMI, and compared with White individuals, Asian individuals had significantly lower odds of referral (OR=0.78, 95% CI 0.65 to 0.94), as did Black individuals (OR=0.56, 95% CI 0.41 to 0.79). There was no clear evidence of a difference for individuals of Mixed ethnicity, or for those classified as Other or Unknown ethnicity. BMI was also independently associated with referral. Compared with individuals at normal BMI, underweight individuals were more likely to be referred (OR=1.81, 95% CI 1.67 to 1.97), while those who were overweight (OR=0.71, 95% CI 0.64 to 0.78) or obese class I (OR=0.79, 95% CI 0.70 to 0.89) were less likely to be referred; there was no clear evidence of a difference for obese class II+ (OR=1.06, 95% CI 0.96 to 1.17).

**Figure 1:**
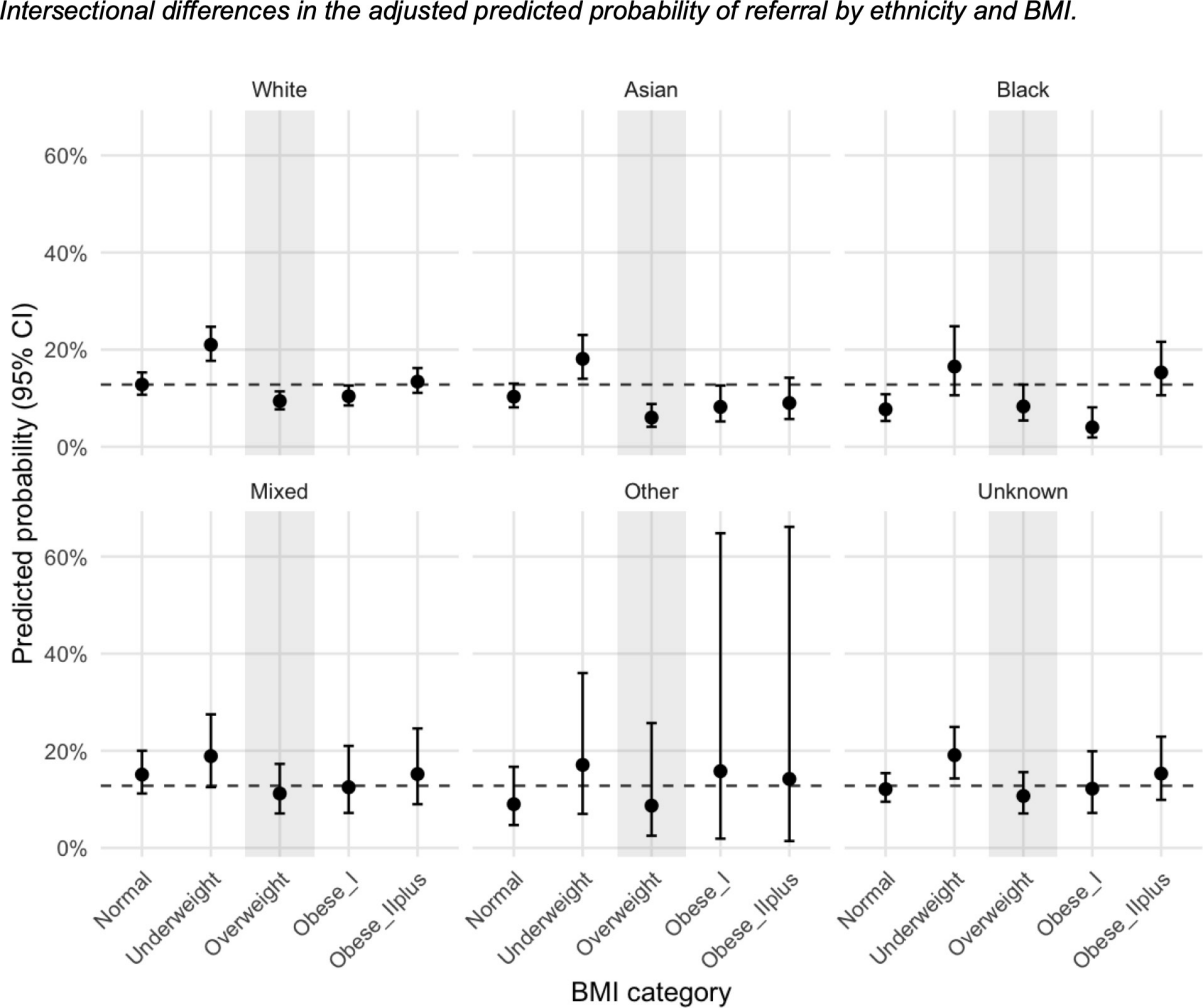
Adjusted predicted probabilities (95% CI) are shown for each ethnicity–BMI combination. The shaded column denotes the overweight BMI category, where referral probabilities were consistently lower across ethnic groups. The dashed horizontal line represents the predicted probability for White individuals at normal BMI (reference category). BMI, body mass index.

There was little evidence that the association between ethnicity and referral varied across BMI categories, as most interaction terms were not statistically significant, indicating that ethnic differences in referral were largely consistent across BMI groups. The only clear evidence of interaction was observed for Black individuals in the Obese II+BMI category, who had higher odds than would be expected based on the separate Black and BMI effects (OR=2.05, 95% CI 1.24 to 3.41). These results are summarised in table 6 and figure 1. Adjusted predicted probabilities and risk differences in ED referrals by BMI×ethnicity are presented in online supplemental SM 5.1–SM 5.3. In a separate adjusted model, referral probability differed significantly by region (p<0.001), with the highest referral probabilities observed in the North West and South East and the lowest in the North East (SM 5.4).

**Table 6:** Association between ethnicity, BMI and referral to ED services (n=32 187)

Variable	OR	95% CI	P value
Ethnicity (ref=white)			
Asian	0.78	0.65–0.94	0.009
Black	0.56	0.41–0.79	<0.001
Mixed	1.21	0.92–1.61	0.179
Other	0.68	0.34–1.33	0.257
Unknown	0.94	0.76–1.16	0.057
BMI category (ref=normal)			
Underweight	1.81	1.67–1.97	<0.001
Overweight	0.71	0.64–0.78	<0.001
Obese I	0.79	0.70–0.89	<0.001
Obese II+	1.06	0.96–1.17	0.264
Ethnicity×BMI interaction			
Asian×underweight	1.06	0.79–1.44	0.689
Black×underweight	1.32	0.74–2.35	0.356
Mixed×underweight	0.72	0.42–1.23	0.232
Unknown×underweight	0.94	0.66–1.35	0.748
Asian×overweight	0.79	0.53–1.19	0.266
Black×overweight	1.55	0.89–2.70	0.117
Mixed×overweight	1.00	0.58–1.74	0.988
Other×overweight	1.36	0.32–5.78	0.680
Unknown×overweight	1.22	0.77–1.93	0.388
Asian×Obese I	0.98	0.60–1.61	0.946
Black×Obese I	0.63	0.28–1.41	0.263
Mixed×Obese I	1.02	0.53–1.97	0.954
Other×Obese I	2.39	0.22–25.91	0.472
Unknown×Obese I	1.28	0.71–2.31	0.413
Asian×Obese II+	0.82	0.49–1.37	0.446
Black×Obese II+	2.05	1.24–3.41	0.005
Mixed×Obese II+	0.95	0.50–1.80	0.880
Other×Obese II+	1.58	0.12–20.26	0.727
Unknown×Obese II+	1.24	0.75–2.06	0.408

ORs and 95% CIs are from a multivariable logistic regression model with ever receiving an eating disorder referral as the outcome. Cases included in the cohort because of an ED referral, but with no ED diagnosis, were excluded from this analysis. The model includes an interaction between ethnicity and BMI category and adjusts for age, sex, ED diagnosis, IMD quintile and smoking status. White ethnicity and normal BMI are the reference categories.

BMI, body mass index; ED, eating disorder; IMD, Index of Multiple Deprivation.

## Discussion

### Main findings

In this large, matched cohort study using primary care data, individuals with EDs had substantially higher primary care contacts compared with controls across total, face-to-face, and telephone or virtual types of contact. Contact rates were elevated across all ED diagnoses, with the highest rates observed for individuals with ARFID, BED and mixed ED presentations, with broadly similar findings in BMI-adjusted sensitivity analyses. Individuals from minoritised ethnic groups had consistently lower rates of contact compared with White individuals. In adjusted referral models, individuals from Black and Asian ethnic groups were less likely to be referred to specialist ED treatment, even when we accounted for diagnosis and BMI.

### The meaning of the study

The present study findings add to a growing literature of studies suggesting low rates of referral to treatment for individuals with EDs but high rates of attendance in primary care.^3 7^ This is consistent with evidence that individuals with EDs have higher comorbidities of both physical and mental health problems compared with individuals without EDs which is likely to impact primary care use.^1 3^ These findings should also be considered in light of evidence showing that comorbid physical health problems are consistently cited as facilitators of help-seeking for EDs.^31 32^

Despite high levels of primary care contact, fewer than one quarter of cases in our sample received a referral to specialist ED services. These findings are consistent with epidemiological evidence showing that referrals from primary care remain low, even when individuals present with diagnostic threshold ED symptoms.^7 33^ The results are also consistent with qualitative accounts in which individuals and carers describe repeated consultations before ED referral, or report being dismissed.^34 35^ They may also reflect limited service capacity in secondary care, long wait times, or referral thresholds (eg, BMI cut-offs), as GPs may be less likely to refer when there is no clear treatment pathway or when they anticipate that the referral will be rejected.^35 36^

The present study also extends existing research examining patterns of reduced primary care use and help-seeking behaviour among individuals from minoritised ethnic groups.^37^ Evidence suggests that clinician confidence in identifying EDs in individuals from minoritised groups, including ethnic minorities, is low.^38^ This is likely to negatively affect patient experiences in primary care, including feelings of being dismissed or disparities in treatment access and experiences.^37^ Drawing on broader healthcare utilisation research, there are also factors which may contribute to lower use of primary care services among individuals from minoritised ethnic backgrounds. These include stigma, beliefs about self-reliance and mistrust of clinicians and healthcare systems.^37^ Taken together, both patient-related and clinician-related factors may influence primary care use, help-seeking for EDs, and subsequent access to specialised ED treatment among individuals from minoritised ethnic backgrounds.

Although OSFED has been reported to represent a substantial proportion of ED presentations in community and clinical populations,^39^ relatively few individuals in our cohort received a specific OSFED diagnosis (6.6%). This may partly reflect the study observation period spanning both Diagnostic and Statistical Manual of Mental Disorders Fourth Edition (DSM-IV) and Diagnostic and Statistical Manual of Mental Disorders Fifth Edition (DSM-5) diagnostic classifications, with OSFED only introduced following publication of DSM-5.^40^ Instead, a large proportion of participants either had no recorded ED diagnosis despite referral to specialist services or had a non-specific ‘Eating Disorder’ diagnostic code. This pattern is consistent with previous CPRD-based ED studies and may reflect challenges distinguishing between ED presentations in routine primary care settings.^1^ Notably, individuals with OSFED and ED-unspecified diagnoses demonstrated higher primary care consultation rates to those with AN and BN, potentially reflecting challenges identifying ED presentations outside of AN and BN or differences in how these presentations are managed in primary care.^41^ Furthermore, a relatively high proportion of individuals in our cohort were diagnosed with AN (43%), which may reflect both the medical risk associated with AN and its clinical visibility, with individuals more likely to present earlier to healthcare services and require hospitalisation compared with other ED presentations.^42^

The results from this study indicated that referral rates were higher than expected for Black individuals with high BMI (Obese II+) compared with the results from ethnicity or BMI separately. This is interesting in the context of existing evidence that Black individuals presenting for treatment of EDs often have higher BMI and more severe eating-related concerns compared with community counterparts.^43^ However, this pattern may also reflect differences in diagnostic presentation, as BED, which is typically associated with higher BMI, is relatively more common among Black individuals with EDs.^44^ Framing these findings in relation to diagnostic profiles, rather than BMI alone, is important given that overall rates of treatment for EDs remain low among Black individuals, regardless of weight status.^43 45^

Individuals with a diagnosis of ARFID showed the highest consultation rates compared with non-ED controls in this study. This may be influenced by limited treatment pathways for ARFID in the UK, where fewer than one-third (29%) of specialist ED services are commissioned to provide ARFID care.^36 46^ It may also reflect the association between ARFID and gastrointestinal symptoms, as well as its earlier age of onset (mean 10.8–11.2 years) compared with other EDs, which may contribute to greater healthcare contact before diagnosis.^30 47^

### Strengths/weaknesses of the study

This is the first UK study to examine primary care contacts by individuals with EDs. Using this large, representative dataset provided rich demographic information and allowed examination of differences in service use for individuals based on demographic characteristics. It also answers existing calls to examine the experiences of individuals from minoritised ethnic groups with EDs and to include a wider range of ED diagnoses, not just BN and AN.^4 38^ As we used routinely collected EHR data, we were able to bridge the gap between experiences of individuals in secondary care (eg, specialist ED treatment) and community populations. Using a large representative sample makes this work generalisable to individuals accessing primary care in England.

The current study is limited by differences in coding practices for individual clinicians (GPs), which may impact the consistency and accuracy of ED diagnoses and consultation events. For example, ‘anorexia’ was retained in the diagnostic codelist to avoid missing cases where it was used as a shorthand for AN, however, the term may also be used more generally to indicate loss of appetite. Inclusion of this code may have resulted in some individuals being classified as having AN despite not having an ED diagnosis. The implication of this potential misclassification is that our results may underestimate the true differences between cases and controls. In addition, the study spans several diagnostic classification frameworks for EDs including DSM-IV, DSM-5, ICD-10 and ICD-11. The introduction of the DSM-5 and ICD-11 broadened ED diagnostic criteria and may have increased the number of individuals eligible for diagnosis over time, though this is unlikely to substantially affect the study’s primary focus examining demographic differences in healthcare use. Furthermore, the EHR data we accessed for this study only includes those who were registered with a GP and who presented to primary care. This means that we are missing data for individuals who do not have access to primary care, those who move frequently and thus have changed GP practices (eg, university students) or those who do not seek help at all. There would also be individuals with ED symptoms who are attending primary care but have not received an ED diagnosis and, therefore, their experiences are not captured here. For pragmatic reasons, we were unable to fully examine all observation codes linked to individual consultation events and we do not have access to clinical data written by the GP about their encounter. This means we cannot be certain of the clinical nature of each consultation including information about the severity of ED symptoms, clinician identified links between ED symptoms and other health problems, or times when referrals for specialist ED treatment were offered but declined by individual patients. Additionally, cultural and linguistic differences in how symptoms are described and communicated during healthcare encounters may also influence recognition, clinical diagnosis and referral decisions across ethnic groups.^48^

Our study did not include access to linked secondary care data (eg, HES or Mental Health Services Data Set) and so we were also unable to determine whether referral events logged in primary care were accepted by specialist ED services or resulted in treatment. This may be particularly important as, in the UK, one in seven NHS patients referred from primary to secondary care services fall into a ‘referral black hole’, where referrals are delayed or rejected, highlighting potential gaps in care even after the referral has been initiated.^49^

Over the study period, changes to diagnostic classification of EDs in the DSM-5 (2013) and the introduction of the ICD-11 (2022) may have influenced diagnoses or proportion of diagnoses included in the sample. Practice-level IMD quintile data were used as a proxy for socioeconomic status, meaning that individual factors such as employment could not be included as potential confounders in the present analysis.

Finally, the ethnic categories used in this study grouped diverse subgroups into broad classifications (eg, combining individuals from Indian and Chinese backgrounds as ‘Asian’), potentially obscuring important differences in healthcare experiences. For example, individuals from Asian sub-groups have unique migration histories and cultural attitudes towards healthcare which would impact patterns in use of primary care services and help-seeking for EDs.^50^ As a result, these findings may mask substantial heterogeneity within Asian and Black ethnic groups and should be interpreted with caution.

### Implications for clinicians and policy

Two key unmet needs identified are amenable to clinical intervention. First, individuals from minoritised ethnic groups had fewer primary care contacts, which may reflect barriers previously described in the literature, including stigma, norms of self-reliance and reduced trust in healthcare systems.^37^ Second, even after adjustment for diagnosis and BMI, individuals from minoritised ethnic groups were less likely to be referred to specialist ED services, suggesting a potential provider-level barrier. These findings indicate a need for increasing awareness of EDs within minoritised communities, alongside culturally inclusive adaptations in primary care to support help-seeking.^51^ They also highlight the potential value of targeted GP education to improve screening and recognition of EDs across diverse populations as physician knowledge about EDs can be low, and improving screening procedures in primary care has been demonstrated to be effective outside the UK.^38 52^

### Unanswered questions and future research

Future research should examine whether the types of primary care contacts differ for individuals with EDs. Previous studies suggest that individuals with EDs may be more likely to seek care for gastrointestinal or gynaecological concerns, and it would be valuable to assess whether these patterns are also observed in UK primary care data and whether these patterns vary by ethnic background or ED diagnosis.^53^ It could also be informative to explore whether consultation patterns differ according to ED severity, although severity is not routinely coded within CPRD Aurum and may require the development of proxy indicators using clinical comorbidities. Furthermore, linking primary care records with secondary care HES would move beyond patterns of presentation to examine whether referrals result in specialist assessment and treatment in secondary care. Our study was restricted to adults, although evidence indicates that EDs typically onset during adolescence, and consequently examination of primary care use among individuals under 18 years remains an important area for future research. The present study focused on the 2-year period prior to a diagnosis or referral, and it would be useful to examine whether these patterns persist or change in primary care in the period after diagnosis and/or treatment.^5^ As analyses were restricted to data from England and a small number of practices in Northern Ireland, future studies incorporating GP practices across all UK nations would help assess the generalisability of these findings. Finally, it would be useful to have a more nuanced examination of the patterns of primary care use and primary care experiences of individuals within distinct sub-groups of the minoritised ethnic groups that we examined.

This study provides important evidence on patterns of primary care use among individuals with EDs prior to diagnosis or referral. Given the central role of primary care in enabling timely access to effective treatment, these findings highlight the need to address both patient- and provider-level barriers to ED help-seeking within primary care settings, particularly for minoritised ethnic groups.

## Supplementary material

10.1136/bmjopen-2026-119143 online supplemental file 1

## Data Availability

Data may be obtained from a third party and are not publicly available.
