# Supplementary material for: Primary care use among adults with eating disorders in England: a population-based cohort study using electronic health records
Source: BMJ Open. 2026 Jun 24;16(6):e119143. doi: 10.1136/bmjopen-2026-119143 (PMC13295994; doi:10.1136/bmjopen-2026-119143)
Supplement: online supplemental file 1 [file bmjopen-16-6-s001.pdf]

Supplementary Materials to: "Primary care use among adults with eating disorders in England: a population-based cohort study using electronic health records"

Supplementary Materials 1: STROBE checklist and RECORD statement

Supplementary Materials 2: Codelists and Checklists for variables

SM 2.1: ED Diagnosis

SM 2.2: BMI

SM 2.3: Hypokalaemia

SM 2.4: Referral events

SM 2.5: Consultation codelists

Supplementary Materials 3: Missing Data

Supplementary Materials 4: Sensitivity Analyses

SM 4.1: Sensitivity analysis by smoking data inclusion

SM 4.2: Sensitivity analysis by BMI

Supplementary Materials 5: Intersectional quantitative analyses

SM 5.1: Adjusted predicted probabilities of being referred by ethnicity x BMI

SM 5.2: Risk differences in ED referral by ethnicity within BMI category

SM 5.3: Risk differences in ED referral by BMI category within ethnicity

SM 5.4: Adjusted probability of eating disorder referral by UK region

Supplementary Materials 1 – STROBE Checklist (Cohort Studies)

	Item No	Recommendation	Page No
**Title and Abstract**	1	(a) Indicate the study's design with a commonly used term in the title or the abstract	1-2
		(b) Provide in the abstract an informative and balanced summary of what was done and what was found	2-3
**Introduction**			
Background/rationale	2	Explain the scientific background and rationale for investigation being reported	5-6
Objectives	3	State specific objectives, including any prespecified hypotheses	6-7
**Methods**			
Study Design	4	Present key elements of the study design early in the paper	7
Setting	5	Describe the setting, locations, and relevant dates, including periods of recruitment, exposure, follow-up, and data collection	2; 7-8
Participants	6	(a) Give the eligibility criteria, and the sources and methods of case ascertainment and control selection. Give the rationale for the choice of cases and controls.	7-8
		(b) For matched studies, give matching criteria and the number of controls per case	7-8
Variables	7	Cleary define all outcomes, exposures, predictors, potential confounders, and effect modifiers. Give diagnostic criteria, if applicable	8-11<br>Table 1
Data sources/measurement	8*	For each variable of interest, give sources of data and details of methods of assessment (measurement). Describe comparability of assessment methods if there is more than one group	7-11, Table 1
Bias	9	Describe any efforts to address potential sources of bias	8; 10-13; 23-26
Study Size	10	Explain how the study size was arrived at	7-8
Quantitative variables	11	Explain how quantitative variables were handled in the analyses. If applicable, describe which groupings were chosen and why	8-13; Table 1
Statistical methods	12	(*a*) Describe all statistical methods, including those used to control for confounding	11-13
		(b) Describe any methods used to examine subgroups and interactions	12-13
		(c) Explain how missing data were addressed	13<br>SM3
		(d) If applicable, explain how matching of cases and controls was addressed	8; 11-12
		(e) Describe any sensitivity analyses	12-13<br>SM 4.1-4.2
**Results**			
Participants	13*	(a) Report numbers of individuals at each stage of study—eg numbers potentially eligible, examined for eligibility, confirmed eligible, included in the study, completing follow-up, and analysed	13-15; Table 2
		(b) Give reasons for non-participation at each stage	NA
		(c) Consider use of a flow diagram	NA

Descriptive data	14*	(a) Give characteristics of study participants (eg demographic, clinical, social) and information on exposures and potential confounders	13-15 Table 2
		(b) Indicate number of participants with missing data for each variable of interest	SM3
Outcome data	15*	Report numbers of outcome events or summary measures over time.	15-21; Tables 3-6; Figure 1
Main results	16	(a) Give unadjusted estimates and, if applicable, confounder-adjusted estimates and their precision (eg, 95% confidence interval). Make clear which confounders were adjusted for and why they were included	15-21; Tables 3-6
		(b) Report category boundaries when continuous variables were categorized	p. 9-10; Table 1; Table 2
		(c) If relevant, consider translating estimates of relative risk into absolute risk for a meaningful time period	Figure 1, SM 5.1-5.4
Other analyses	17	Report other analyses done—eg analyses of subgroups and interactions, and sensitivity analyses	12-21 SM 4.1-4.2; SM 5.1-5.4
**Discussion**			
Key Results	18	Summarise key results with reference to study objectives	21-22
Limitations	19	Discuss limitations of the study, taking into account sources of potential bias or imprecision. Discuss both direction and magnitude of any potential bias	23-26
Interpretation	20	Give a cautious overall interpretation of results considering objectives, limitations, multiplicity of analyses, results from similar studies, and other relevant evidence	22-23; 26-27
Generalisability	21	Discuss the generalisability (external validity) of the study results	24-26
**Other Information**			
Funding	22	Give the source of funding and the role of the funders for the present study and, if applicable, for the original study on which the present article is based	27

*Give information separately for cases and controls

Note: An Explanation and Elaboration article discusses each checklist item and gives methodological background and published examples of transparent reporting. The STROBE checklist is best used in conjunction with this article (freely available on the Web sites of PLoS Medicine at http://www.plosmedicine.org/, Annals of Internal Medicine at http://www.annals.org/, and Epidemiology at http://www.epidem.com/). Information on the STROBE Initiative is available at http://www.strobe-statement.org.

The RECORD statement – checklist of items, extended from the STROBE statement, that should be reported in observational studies using routinely collected health data.

	Item No.	STROBE items	Location in manuscript where items are reported	RECORD items	Location in manuscript where items are reported
**Title and abstract**	**1**	(a) Indicate the study's design with a commonly used term in the title or abstract (b) Provide in the abstract an informative and balanced summary of what was done and what was found	p. 1-3	**Record 1.1:** The type of data used should be specified in the title or abstract. When possible, the name of the databases used should be included.	p. 1-2
				**Record 1.2:** If applicable, the geographic region and timeframe within which the study took place should be reported in the title or abstract.	p. 1-2
				**Record 1.3:** If linkage between databases was conducted for the study, this should be clearly stated in the title or abstract.	p. 2
**Introduction**					
	**2**	Explain the scientific background and rationale for the investigation before reported	p. 5-6		
	**3**	State specific objectives, including any prespecified hypotheses	p. 6-7		
**Methods**					
**Study Design**	**4**	Present key elements of study design early in the paper	p. 7		
**Setting**	**5**	Describe the setting, locations, and relevant dates, including periods of recruitment, exposure, follow-up, and data collection	p. 2; p. 7-8		
**Participants**	**6**	(a). *Cohort Study* – Give the eligibility criteria, and the sources and methods of selection of participants. Describe methods of follow-up.	p. 7-8	**Record 6.1:** The methods of study population selection (such as codes or algorithms used to identify subjects) should be listed in detail. If this is not possible, an explanation should be	p. 7<br>Table 1

		Case control study – Give the eligibility criteria, and the sources and methods of case ascertainment and control selection. Give the rationale for the choice of cases and controls.<br>*Cross-sectional study* – Give the eligibility criteria, and the sources and methods of selection of participants<br><br>(b). *Cohort study* – For matched studies, give matching criteria and number of exposed and unexposed<br>*Case-control study* – For matched studies, give matching criteria and the number of controls per case		provided.<br><br>**Record 6.2:** Any validation studies of the codes or algorithms used to select the population should be referenced. If validation was conducted for this study and not published elsewhere, detailed methods and results should be provided.<br><br>**Record 6.3:** If the study involved linkage of databases, consider use of a flow diagram or other graphical display to demonstrate the data linkage process, including the number of individuals with linked data at each stage.	 <br>Table 1 p. 11-12<br><br><br><br><br><br>Table 1<br>SM 3
**Variables**	**7**	Clearly define all outcomes, exposures, predictors, potential confounders, and effect modifiers. Give diagnostic criteria, if applicable.	p. 8-11, Table 1	**Record 7.1:** A complete list of codes and algorithms used to classify exposures, outcomes, confounders, and effect modifiers should be provided. If these cannot be reported, an explanation should be provided.	Table 1<br>SM 2.1-2.5
**Data sources/measurement**	**8**	For each variable of interest, give sources of data and details of methods of assessment (measurement). Describe comparability of assessment methods if there is more than one group	p. 7-11, Table 1		
**Bias**	**9**	Describe any efforts to address potential sources of bias	p. 11-13; p. 23-26		
**Study Size**	**10**	Explain how the study size was arrived at	p. 7-8		
**Quantitative variables**	**11**	Explain how quantitative variables were handled in the analyses. If applicable, describe which groupings were chosen and why	p. 8-13; Table 1		

**Statistical Methods**	**12**	(a) Describe all statistical methods, including those used to control for confounding<br>(b) Describe any methods used to examine subgroups and interactions<br>(c) Explain how missing data were addressed<br>(d) *Cohort study* – If applicable, explain how loss to follow-up was addressed<br>*Case control* – If applicable, explain how matching of cases and controls was addressed<br>*Cross-sectional study* – If applicable, describe analytical methods taking account of sampling strategy<br>(e) Describe any sensitivity analyses	p.. 11-13; SM 3; SM 4.1-4.2; SM 5.1-5.4		
**Data access and cleaning methods**				**Record 12.1:** Authors should describe the extent to which the investigators had access to the database population used to create the study population	p. 7-8; Table 1
				**Record 12.2:** Authors should provide information on the data cleaning methods used in the study	p. 11-13
**Linkage**				**Record 12.3:** State whether the study included person -level, institutional -level, or other data linkage across two or more databases. The methods of linkage and methods of linkage quality evaluation should be provided.	Table 1
**Results**					
**Participants**	**13**	(a) Report the numbers of individuals at each stage of the study (e.g. numbers potentially eligible, examined for eligibility, confirmed eligible, included in	p. 13-15; Table 2	**Record 13.1:** Describe in detail the selection of the persons included in the study (i.e., study population selection) including filtering based on data	p. 8

		the study, completing follow-up, and analysed)<br>(b) Give reasons for non-participation at each stage.<br>(c) Consider use of a flow diagram		quality, data availability and linkage. The selection of included persons can be described in the text and/or by means of the study flow diagram.	
**Descriptive data**	**14**	(a) Give characteristics of study participants (e.g., demographic, clinical, social) and information on exposures and potential confounders (b) Indicate the number of participants with missing data for each variable of interest (c) Cohort study - summarise follow -up time (e.g., average and total amount)	p. 13-15; Table 2; SM 3		
**Outcome data**	**15**	*Cohort study* - Report numbers of outcome events or summary measures over time<br>*Case -control study* - Report numbers in each exposure category, or summary measures of exposure<br>*Cross -sectional study* - Report numbers of outcome events or summary measures	p. 15-21; Tables 3-6; Figure 1		
**Main results**	**16**	(a) Give unadjusted estimates and, if applicable, confounder - adjusted estimates and their precision (e.g., 95% confidence interval). Make clear which confounders were adjusted for and why they were included (b) Report category boundaries when continuous variables were categorized (c) If relevant, consider translating estimates of relative risk into absolute risk for a	p. 15-21; Tables 3-6; Figure 1; SM 5.1-5.4		
**Other analyses**	**17**	Report other analyses done<br>—<br>e.g., analyses of subgroups and interactions, and sensitivity analyses	p. 12-13; p. 17-21; SM 4.1-4.2; SM 5.1-5.4		
**Discussion**					

**Key Results**	**18**	Summarise key results with reference to study objectives	p. 21-22		
**Limitations**	**19**		p. 23-26	**Record 19.1:** Discuss the implications of using data that were not created or collected to answer the specific research question(s). Include discussion of misclassification bias, unmeasured confounding, missing data, and changing eligibility over time, as they pertain to the study being reported.	p. 26-27
**Interpretation**	**20**	Give a cautious overall interpretation of results considering objectives, limitations, multiplicity of analyses, results from similar studies, and other relevant evidence	p. 22-23; p. 26-27		
**Generalisability**	**21**	Discuss the generalisability (external validity) of the study results	p. 24-26		
**Other Information**					
**Funding**	**22**	Give the source of funding and the role of the funders for the present study and, if applicable, for the original study on which the present article is based	p. 30		
**Accessibility of protocol, raw data, and programming code**				**RECORD 22.1:** Authors should provide information on how to access any supplemental information such as the study protocol, raw data, or programming code.	p. 3

*Reference: Benchimol EI, Smeeth L, Guttmann A, Harron K, Moher D, Petersen I, Sørensen HT, von Elm E, Langan SM, the RECORD Working Committee. The REporting of studies Conducted using Observational Routinely-collected health Data (RECORD) Statement. PLoS Medicine 2015; in press.

*Checklist is protected under Creative Commons Attribution (CC BY) license.

Supplementary Materials 2.1 Eating Disorder Diagnosis Codelist and Checklist

ED Diagnosis Codelist

medcodeid	term
251631011	H/O: anorexia nervosa
317233013	[D]Anorexia
317235018	[D]Anorexia NOS
3423851000006118	AN - Anorexia nervosa
3531021000006113	Anorexia nervosa, binge-eating purging type
363321000006111	[X]Anorexia nervosa
366521000006113	Atypical anorexia nervosa
3763791000006115	Anorexia nervosa, restricting type
3763801000006119	Anorexia nervosa, restricting subtype
404431014	Appetite loss - anorexia
94597012	Anorexia nervosa
960281000006115	Anorexia
1222496015	Bulimia
3027091000006119	Bulimia nervosa, purging type
3468921000006116	Bulimia nervosa, nonpurging type

366541000006118	Atypical bulimia nervosa
368051000006111	Bulimia nervosa
368061000006113	[X]Bulimia NOS
3769021000006116	BN - Bulimia nervosa
527421000006116	Bulimia (non-organic overeating)
295436010	Other and unspecified non-organic eating disorders
295437018	Unspecified non-organic eating disorder
295451018	Other specified non-organic eating disorder
295452013	Non-organic eating disorder NOS
296379015	[X]Eating disorder, unspecified
401889011	[X]Other eating disorders
882611000006115	Eating disorder NOS - psychog.
988931000006112	Eating disorder NOS - psychog.
370419010	Binge Eating Disorder
72301010000061	Binge eating disorder
370419010	Binge eating
14532571000006	Binge eating behaviour
417851000006113	Overeating associated with other psychological disturbances

577901000006119	Compulsive eating disorder
3515759010	Insufficient intake of food and water with self neglect
7532211000006110	Avoidant restrictive food intake disorder
1620911000006112	Eating disorders
296361019	Eating disorder
8440231000006115	Eating disorder co-occurrent with diabetes mellitus type 1
23968015	Pica

Checklist – Matthewman et al. (2024)

	Step No.	Item	Information to be provided	Response
		**Metadata**		
Metadata	0	a. Name	*What is the name of the codelist?*	Eating Disorder Codelist
		b. Author(s)	*Who created the codelist?*	Jessica Wilkins, Karina Allen, Ulrike Schmidt, Alex Dregan
		c. Date finalised	*When was the codelist finalised?*	June 2024
		d. Target data source	*What data is the codelist designed to be used with?*	Clinical Practice Research Datalink. (2025). CPRD Aurum June 2025 (Version 2025.06.001) [Data set]. Medicines and Healthcare products Regulatory Agency. https://doi.org/10.48329/a94w-e055

		e. Terminology	*What is the terminology? (e.g., SNOMED, ICD)*	MedCodeID and Term
	**Define a clinical concept**			
Define	1	a. Concept	*What is the clinical concept (e.g., the disease, drug, test result, etc…) of interest?*	All eating disorder diagnoses which appear in DSM-IV, DSM-V and the ICD-10. Including anorexia nervosa, atypical anorexia nervosa, bulimia nervosa, atypical bulimia nervosa, binge eating disorder, otherwise specified feeding and eating disorder (also eating disorder not otherwise specified), avoidant restrictive food intake disorder, and pica.
		b. Timeframe	*Should the codelist capture new, current, and/or previous events?*	New, current, and/or previous events
		c. Accuracy	*Should the codelist capture probable or definite codes?*	Probable and/or definite codes
		d. Setting	*What is the (health care) setting (e.g., primary care, hospital care)?*	Primary care
**Identify and evaluate existing codelists**				
Search	2	a. Sources searched	*Which sources were searched (e.g., internet search, codelist repositories)?*	We used this codelist repository: https://clinicalcodes.rss.mhs.man.ac.uk/

		b. Existing codelists found	*Which suitable codelists did you find?*	https://clinicalcodes.rss.mhs.man.ac.uk/medcodes/article/76/codelist/res76-eating-disorders/
<u>Verify</u>	3	a. Verified by others	*Which information is available to verify the quality of suitable codelists?*	The codelist will be published in the codelist repository: https://clinicalcodes.rss.mhs.man.ac.uk/ and also be available via supplementary materials to be reviewed and replicated by others.
		b. Verified by yourself	*Which checks did you conduct to verify the quality of suitable codelists?*	Members of our research term include clinicians who work in eating disorders including a psychiatrist, clinical psychologist and counselling psychologist all of whom reviewed the codelist. We verified the quality of this suitable codelist by reviewing the original paper and the means by which study authors verified their codelist.
<u>Reference</u>	4	a. Existing codelists used	*Are you making use of any existing codelists? If yes, reference these, and specify how they are being used.*	We used this codelist: https://clinicalcodes.rss.mhs.man.ac.uk/medcodes/article/76/codelist/res76-eating-disorders/ to see if we captured the same codes in our codelist as the list above. However, it is important to note that not all codes used in the list above will be used for the purposes of our study.
		**Create a new codelist**		
<u>Prepare</u>	5	a. Synonyms	*What are synonyms and related words for the clinical concept (e.g., different names for a disease/drug) and how did you identify these (e.g., source of clinical knowledge)?*	Synonyms and related words to capture eating disorder diagnosis include:<br>• Eating disorders<br>• Non-organic eating disorder<br>• Appetite loss<br>• Overeating<br>• Insufficient intake of food and water with self-neglect<br>• Compulsive eating disorder<br><br>These synonyms were identified through close consultation with an experienced study team:<br>• Dr. Jamie Scuffell - Population health epidemiologist and General Practitioner

				• Professor Ulrike Schmidt – Consultant Psychiatrist and Professor of Eating Disorders. Director of the Centre for Research on Eating and Weight Disorders at King's College London<br>• Dr. Karina Allen - Consultant Clinical Psychologist in the outpatient adult eating disorder service at South London and Maudsley NHS Foundation Trust<br>• Ms. Jessica Wilkins - PhD student and HCPC registered counselling psychologist at King’s College London
		b. Exceptions	*What should not be included in the codelist?*	Any codes related to a referral for eating disorder treatment/to clinic as these were included in a separate eating disorder referral code list (also available in supplementary materials).
Create	6	a. Method used	*Which method (e.g., a script, a tool) did you use to create the draft codelist?*	Exporting search results from the Clinical Practice Research Datalink Aurum code browser. Script developed in R to search for medical code terms in the extracted dataset from Aurum to triangulate with search results from the code browser and from existing codelists.
		b. Search terms	*Which search terms, and if applicable, exclusion terms did you use?*	CPRD aurum code browser<br>Search terms used:<br>• “eating disorder*”<br>• “*anorexi*”<br>• “*bulimi*”<br>• “ARFID”<br>• “avoidant restrictive food*”<br>• “*binge eat*”<br>• “BED”<br>• “*eating disorder*”<br>• “insufficient intake*”<br>• “PICA”<br>• “otherwise specified food*”

		c. Hierarchy used to extend search	*Did you use a dictionary hierarchy (e.g., ICD-10 chapters, SNOMED-CT concepts) to modify your search? If yes, specify.*	No
		d. Decisions made while iterating	*Which decisions did you make while iteratively refining the draft codelist?*	Exclude <1000 observations<br>Exclude unclear symptoms e.g. "vomiting"
		e. (Optional) Categories	*Did you specify subcategories within the codelist? If yes, specify.*	Yes<br>Diagnostic categories were created for analysis:<br>Anorexia Nervosa, Bulimia Nervosa, BED, OSFED, ARFID, PICA, unspecified eating disorder.
	**Review, finalise and publish**			
<u>Review</u>	7	a. Reviewers	*Who reviewed the codelist and what expertise did reviewers have?*	This codelist was verified by the following individuals:<br>• Dr. Jamie Scuffell –Population health epidemiologist and General Practitioner<br>• Professor Ulrike Schmidt - Consultant Psychiatrist and Professor of Eating Disorders. Director of the Centre for Research on Eating and Weight Disorders at King's College London<br>• Dr. Karina Allen - Consultant Clinical Psychologist in the outpatient adult eating disorder service at South London and Maudsley NHS Foundation Trust<br>• Ms. Jessica Wilkins - PhD student and HCPC registered counselling psychologist at King's College London
		b. Scope of review	*What was reviewed (Just the draft codelist or also the method, terms, etc..)?*	The draft codelist was reviewed by Dr. Scuffell, Professor Schmidt, Dr. Allen, and Ms. Wilkins. Ms. Wilkins, Professor Schmidt, and Dr. Allen reviewed methods and terms as well.

		c. Evidence of review	*Where is the review process documented?*	The review process was documented through email exchanges and in research meeting minutes.
Checks	8	a. Internal checks	*What method(s) were used for internal checks, if any, and what are the findings?*	A search was run on the CPRD database to identify eating disorder diagnostic codes which existed in our data set. This list was then checked against our developed codelists and additional variables were added if they appeared in the dataset.
		b. External checks	*What method(s) were used for external checks, if any, and what are the findings?*	Codelists were submitted, along with the study protocol application, for review by CPRD Research Ethics Committee.
Publish	9	a. Codelist published	*Where is the codelist published?*	The codelist will be published in the codelist repository: https://clinicalcodes.rss.mhs.man.ac.uk/

Note. An Explanation and Elaboration article discusses each checklist item and gives methodological background and published examples of transparent reporting. Matthewman J, Andresen K, Suffel A, Lin LY, Schultze A, Tazare J, Bhaskaran K, Williamson E, Costello R, Quint J, Strongman H. Checklist and guidance on creating codelists for routinely collected health data research. NIHR Open Res. 2024 Sep 18;4:20. doi: 10.3310/nihropenres.13550.2. PMID: 39345273; PMCID: PMC11437289.The Codelist checklist is best used in conjunction with this article.

Supplementary Materials 2.2 BMI Codelist and Checklist

medcodeid	term
100716012	Body mass index
1750501000006114	Checking weight monitoring
1780175010	Baseline weight
1808061000006114	Body mass index less than 18.5
1808071000006119	Body mass index 18.5-24.9
1809441000006111	Weight change
1910911000006118	Reported weight
1910931000006112	Reported height
2159952010	Underweight
2160062010	Body mass index 40+ - severely obese
217781000000116	O/E - weight greater than 20% over ideal
2196071000000116	Baseline body mass index
2316211000000111	Unintentional weight gain
2350241000000116	Obese class I (body mass index 30.0 - 34.9)
2350261000000115	Obese class II (body mass index 35.0 - 39.9)
2474325012	Body mass index 20-24 - normal
252191014	Weight increasing
252192019	Weight decreasing
2536041016	Weight static
253669010	Standing height
253672015	O/E -height within 10% average
253673013	O/E-height 10-20% over average
253676017	O/E - height NOS
253677014	Body weight
253679012	O/E -weight 10-20% below ideal
253680010	O/E - weight within 10% ideal
253687013	O/E - Underweight
253688015	O/E - weight NOS
253844013	Normal body mass index
253845014	Increased body mass index
253846010	Decreased body mass index
253847018	Body mass index index 25-29 - overweight
253848011	Body mass index 30+ - obesity

271181000006113	O/E-height > 20% below average
272151000006114	O/E -height > 20% over average
272551000006116	O/E - weight > 20% below ideal
284561000006119	O/E - height 10-20% < average
317244017	Failure to gain weight
356960015	Overweight
402435016	O/E - weight 10-20% over ideal
406169014	Weight steady
411922013	O/E - overweight
451201014	Weight monitoring
453856012	Body mass index less than 20
567001000000116	Counterweight programme
59281000006111	Weight screening
857681000006117	Weight reduction clinic
908721000006111	[RFC] Overweight/ underweight
909921000006118	[RFC] Weight problem
910251000006110	[RFC] Overweight
910261000006112	[RFC] Underweight
923831000006115	O/E - height
923851000006110	O/E - weight
923861000006112	Body mass index
982581000006118	Weight (Over)
982591000006115	Weight (Under)
982601000006111	Height (Over)
982611000006114	Height (Under)
2196071000000116	Baseline body mass index
923861000006112	Body mass index
3484801000006114	BMI - Body mass index
253866018	Height and weight
100716012	Body mass index

Checklist – Matthewman et al. (2024)

	Step	Item	Information to be provided	Response
**Metadata**				
Metadata	0	Name	*What is the name of the codelist?*	BMI Codelist
		Authors	*Who created the codelist?*	Jessica Wilkins, Karina Allen, Ulrike Schmidt
		Date finalised	*When was the codelist finalised?*	November 10, 2025
		Target data source	*What data is the codelist designed to be used with?*	Clinical Practice Research Datalink. (2025). CPRD Aurum June 2025 (Version 2025.06.001) [Data set]. Medicines and Healthcare products Regulatory Agency. https://doi.org/10.48329/a94w-e055
		Terminology	*What is the terminology? (e.g., SNOMED, ICD)*	MedCodeID and Term
**Define a clinical concept**				
Define	1	Concept	*What is the clinical concept (e.g., the disease, drug, test result, etc…) of interest?*	BMI category<br>BMI was categorized as "underweight" if <18.5 kg/m^2, "normal weight" if 18.5-24.9 kg/m^2, "overweight" if 25-29.9 kg/m^2, or "obese" if ≥30 kg/m^2, in accordance with World Health Organization categories (https://www.who.int/data/gho/data/themes/topics/topic-details/GHO/body-mass-index).
		Timeframe	*Should the codelist capture*	New, current, and/or previous events

			new, current, and/or previous events?	
		Accuracy	*Should the codelist capture probable or definite codes?*	Probable and/or definite codes
		Setting	*What is the (health care) setting (e.g., primary care, hospital care)?*	Primary care
**Identify and evaluate existing codelists**				
Search	2	Sources searched	*Which sources were searched (e.g., internet search, codelist repositories)?*	We used this codelist repository: https://clinicalcodes.rss.mhs.man.ac.uk/
		Existing codelists found	*Which suitable codelists did you find?*	https://clinicalcodes.rss.mhs.man.ac.uk/medcodes/article/7/codelist/body_mass_index/
Verify	3	Verified by others	*Which information is available to verify the quality of suitable codelists?*	The codelist will be published in the codelist repository: https://clinicalcodes.rss.mhs.man.ac.uk/ and also be available via supplementary materials to be reviewed and replicated by others.
		Verified by yourself	*Which checks did you conduct to verify the quality of suitable codelists?*	We verified the quality of this suitable codelist by reviewing the original paper and the means by which study authors verified their codelist. In this instance, it was through consultation with Hippisley-Cox and Coupland (2005) that the codes Reeves et al. (2014) used for their replicate study were verified.

Reference	4	Existing codelists used	*Are you making use of any existing codelists? If yes, reference these, and specify how they are being used.*	We used this codelist: https://clinicalcodes.rss.mhs.man.ac.uk/medcodes/article/7/codelist/body_mass_index/ to see if we captured the same codes in our codelist as the list above. However, it is important to note that not all codes used in the list above will be used for the purposes of our study.
**Create a new codelist**				
Prepare	5	Synonyms	*What are synonyms and related words for the clinical concept (e.g., different names for a disease/drug) and how did you identify these (e.g., source of clinical knowledge)?*	Synonyms and related words to capture Body Mass Index (BMI) are:<br>• Weight<br>• Weigh<br>• BMI<br>• Body Mass Index<br>• Height<br>• Weight and Height<br>• Body Mass<br>• Mass<br><br>These synonyms were identified through close consultation with an experienced study team:<br>• Dr. Jamie Scuffell - Population health epidemiologist and General Practitioner<br>• Consultant Psychiatrist and Professor of Eating Disorders. Director of the Centre for Research on Eating and Weight Disorders at King's College London<br>• Dr. Karina Allen - Consultant Clinical Psychologist in the outpatient adult eating disorder service at South London and Maudsley NHS Foundation Trust<br>• Ms. Jessica Wilkins - PhD student and HCPC registered counseling psychologist at King’s College London
		Exceptions	*What should not be included in the codelist?*	Any codes unrelated to weight itself (i.e., no weight-related conditions such as T2DM).
Create	6	Method used	*Which method (e.g., a script, a tool) did you use*	Exporting search results from the Clinical Practice Research Datalink Aurum code browser.

	to create the draft codelist?	
Search terms	*Which search terms, and if applicable, exclusion terms did you use?*	CPRD aurum code browser<br>Search terms used:<br>• *bmi*<br>• *weight*<br>• *mass*<br>• *height*<br>• Body Mass Index<br>• *weigh*<br>• *mass*
Hierarchy used to extend search	*Did you use a dictionary hierarchy (e.g., ICD-10 chapters, SNOMED-CT concepts) to modify your search? If yes, specify.*	No
Decisions made while iterating	*Which decisions did you make while iteratively refining the draft codelist?*	Exclude <1000 observations<br>Exclude unclear weight categories (i.e., O/E, expected weight, ideal weight)
(Optional) Categories	*Did you specify subcategories within the codelist? If yes, specify.*	No
**Review, finalise, and publish**		

Review	7	Reviewers	*Who reviewed the codelist and what expertise did reviewers have?*	This codelist was verified by the following individuals:<br>• Dr. Jamie Scuffell - Population health epidemiologist and General Practitioner<br>• Professor Ulrike Schmidt - Consultant Psychiatrist and Professor of Eating Disorders. Director of the Centre for Research on Eating and Weight Disorders at King's College London<br>• Dr. Karina Allen - Consultant Clinical Psychologist in the outpatient adult eating disorder service at South London and Maudsley NHS Foundation Trust<br>• Ms. Jessica Wilkins - PhD student and HCPC registered counseling psychologist at King's College London
		Scope of review	*What was reviewed (Just the draft codelist or also the method, terms, etc..)?*	The draft codelist was reviewed by Dr. Scuffell, Professor Schmidt, Dr. Allen, and Ms. Wilkins. Ms. Wilkins, Professor Schmidt, and Dr. Allen reviewed methods and terms as well.
		Evidence of review	*Where is the review process documented?*	The review process was documented through email exchanges and research team meeting minutes.
Checks	8	Internal checks	*What method(s) were used for internal checks, if any, and what are the findings?*	A search was run on the CPRD database to identify BMI codes which existed in our data set. This list was then checked against our developed codelists and additional variables were added if they appeared in the dataset.
		External checks	*What method(s) were used for external checks, if any, and what are the findings?*	N/A
Publish	9	Codelist published	*Where is the codelist published?*	The codelist will be published in the codelist repository: https://clinicalcodes.rss.mhs.man.ac.uk/

	Resources published	*Where are the resources used to create the codelist (e.g., scripts, list of terms)?*	We will detail our use of the Clinical Practice Research Datalink Aurum code browser and our use of an existing code list when we publish our codelist in the codelist repository, and highlight these resources in our resulting manuscripts.

Note. An Explanation and Elaboration article discusses each checklist item and gives methodological background and published examples of transparent reporting. Matthewman J, Andresen K, Suffel A, Lin LY, Schultze A, Tazare J, Bhaskaran K, Williamson E, Costello R, Quint J, Strongman H. Checklist and guidance on creating codelists for routinely collected health data research. NIHR Open Res. 2024 Sep 18;4:20. doi: 10.3310/nihropenres.13550.2. PMID: 39345273; PMCID: PMC11437289.The Codelist checklist is best used in conjunction with this article.

Supplementary Materials 2.3 Hypokalaemia Codelist and Checklist

**medcodeid**	**Term**
**493576016**	**Hypokalaemia**
**259000015**	**Electrolytes abnormal**
**1221390012**	**Low serum potassium level**
**253418015**	**Dietary potassium - low**
**144821000006115**	**Serum potassium level**
**3198231000006110**	**Hypokalemia**

Checklist – Matthewman et al. (2024)

	**Step No.**	**Item**	**Information to be provided**	**Response**
		**Metadata**		
Metadata	0	a. Name	*What is the name of the codelist?*	Hypokalaemia Codelist
		b. Author(s)	*Who created the codelist?*	Jessica Wilkins, Karina Allen, Ulrike Schmidt, Jamie Scuffell
		c. Date finalised	*When was the codelist finalised?*	November 2024

		d. Target data source	*What data is the codelist designed to be used with?*	Clinical Practice Research Datalink. (2025). CPRD Aurum June 2025 (Version 2025.06.001) [Data set]. Medicines and Healthcare products Regulatory Agency. https://doi.org/10.48329/a94w-e055
		e. Terminology	*What is the terminology? (e.g., SNOMED, ICD)*	MedCodeID and Term
	**Define a clinical concept**			
Define	1	a. Concept	*What is the clinical concept (e.g., the disease, drug, test result, etc…) of interest?*	Hypokalaemia
		b. Timeframe	*Should the codelist capture new, current, and/or previous events?*	New, current, and/or previous events
		c. Accuracy	*Should the codelist capture probable or definite codes?*	Probable and/or definite codes
		d. Setting	*What is the (health care) setting (e.g., primary care, hospital care)?*	Primary care
	**Identify and evaluate existing codelists**			
Search	2	a. Sources searched	*Which sources were searched (e.g., internet search, codelist repositories)?*	We used this codelist repository: https://clinicalcodes.rss.mhs.man.ac.uk/

		b. Existing codelists found	*Which suitable codelists did you find?*	We did not find any suitable clinical codelists.
<u>Verify</u>	3	a. Verified by others	*Which information is available to verify the quality of suitable codelists?*	The codelist will be published in the codelist repository: https://clinicalcodes.rss.mhs.man.ac.uk/ and also be available via supplementary materials to be reviewed and replicated by others.
		b. Verified by yourself	*Which checks did you conduct to verify the quality of suitable codelists?*	Members of our research term include clinicians who work in eating disorders including a psychiatrist, clinical psychologist and counselling psychologist all of whom reviewed the codelist who used our clinical knowledge to develop search terms. Drafts of the codelist were then shared with a primary care specialist for further verification.
<u>Reference</u>	4	a. Existing codelists used	*Are you making use of any existing codelists? If yes, reference these, and specify how they are being used.*	No.
		**Create a new codelist**		
<u>Prepare</u>	5	a. Synonyms	*What are synonyms and related words for the clinical concept (e.g., different names for a disease/drug) and how did you identify these (e.g., source of clinical knowledge)?*	Synonyms and related words to capture eating disorder referrals include:<br>• Hypopotassemia<br>• Low potassium<br>• Decreased serum potassium<br>• Reduced blood potassium<br><br>These synonyms were identified through close consultation with an experienced study team:<br>• Dr. Jamie Scuffell - Population health epidemiologist and General Practitioner<br>• Professor Ulrike Schmidt - Consultant Psychiatrist and Professor of Eating Disorders. Director of the Centre for Research on Eating and Weight Disorders at King's College London

				• Dr. Karina Allen - Consultant Clinical Psychologist in the outpatient adult eating disorder service at South London and Maudsley NHS Foundation Trust<br>• Ms. Jessica Wilkins - PhD student and HCPC registered counselling psychologist at King's College London
		b. Exceptions	*What should not be included in the codelist?*	Serum potassium codes were only included if associated values were <3.5 mmol/L
<u>Create</u>	6	a. Method used	*Which method (e.g., a script, a tool) did you use to create the draft codelist?*	We exported search results from the Clinical Practice Research Datalink Aurum code browser into an excel file. We also used a script in R to search for medical code terms in the extracted dataset from Aurum to triangulate with search results from the code browser and from existing codelists. Duplicates, identified by medcodeid (not terms) were deleted.
		b. Search terms	*Which search terms, and if applicable, exclusion terms did you use?*	CPRD aurum code browser<br>Search terms used:<br>• *electrolytes*<br>• *hypokalaemia*<br>• *potassium*<br>• "hypopotassemia"
		c. Hierarchy used to extend search	*Did you use a dictionary hierarchy (e.g., ICD-10 chapters, SNOMED-CT concepts) to modify your search? If yes, specify.*	No
		d. Decisions made while iterating	*Which decisions did you make while iteratively refining the draft codelist?*	Terms with <1000 records eliminated.
		e. (Optional) Categories	*Did you specify subcategories within the codelist? If yes, specify.*	No

		**Review, finalise and publish**		
Review	7	a. Reviewers	*Who reviewed the codelist and what expertise did reviewers have?*	This codelist was verified by the following individuals:<br>• Dr. Jamie Scuffell –Population health epidemiologist and General Practitioner<br>• Professor Ulrike Schmidt - Consultant Psychiatrist and Professor of Eating Disorders. Director of the Centre for Research on Eating and Weight Disorders at King's College London<br>• Dr. Karina Allen - Consultant Clinical Psychologist in the outpatient adult eating disorder service at South London and Maudsley NHS Foundation Trust<br>• Ms. Jessica Wilkins - PhD student and HCPC registered counselling psychologist at King’s College London
		b. Scope of review	*What was reviewed (Just the draft codelist or also the method, terms, etc..)?*	The draft codelist was reviewed by Dr. Scuffell, Professor Schmidt, Dr. Allen, and Ms. Wilkins. Ms. Wilkins, Professor Schmidt, and Dr. Allen reviewed methods and terms as well.
		c. Evidence of review	*Where is the review process documented?*	The review process was documented through email exchanges and in research meeting minutes.
Checks	8	a. Internal checks	*What method(s) were used for internal checks, if any, and what are the findings?*	A search was run on the CPRD database to identify hypokalaemia codes which existed in our data set.
		b. External checks	*What method(s) were used for external checks, if any, and what are the findings?*	None.
Publish	9	a. Codelist published	*Where is the codelist published?*	The codelist will be published in the codelist repository: https://clinicalcodes.rss.mhs.man.ac.uk/

b. Resources published	*Where are the resources used to create the codelist (e.g., scripts, list of terms)?*	We will detail our use of the Clinical Practice Research Datalink Aurum code browser and our use of an existing code list when we publish our codelist in the codelist repository, and highlight these resources in our resulting manuscripts.

b. Resources published	*Where are the resources used to create the codelist (e.g., scripts, list of terms)?*	We will detail our use of the Clinical Practice Research Datalink Aurum code browser and our use of an existing code list when we publish our codelist in the codelist repository, and highlight these resources in our resulting manuscripts.

Note. An Explanation and Elaboration article discusses each checklist item and gives methodological background and published examples of transparent reporting. Matthewman J, Andresen K, Suffel A, Lin LY, Schultze A, Tazare J, Bhaskaran K, Williamson E, Costello R, Quint J, Strongman H. Checklist and guidance on creating codelists for routinely collected health data research. NIHR Open Res. 2024 Sep 18;4:20. doi: 10.3310/nihropenres.13550.2. PMID: 39345273; PMCID: PMC11437289.The Codelist checklist is best used in conjunction with this article.

Supplementary Materials 2.4 – Eating Disorder Referral Codelist and Checklist

medcodeid	Term
1780181019	Referral to eating disorders clinic
1764491000006116	Referral to eating disorders outreach clinic
8274831000006110	Eating disorders service
6025191000006115	Eating disorder counselling
6452531000006114	Eating disorders management
7567401000006119	Eating disorder clinic
664031000000110	Seen in eating disorder clinic
1664151000006114	Seen in eating disorders clinic
245171100000111	Eating disorder care plan

Checklist – Matthewman et al. (2024)

	Step No.	Item	Information to be provided	Response
		**Metadata**		
Metadata	0	a. Name	*What is the name of the codelist?*	Eating Disorder Referral Codelist
		b. Author(s)	*Who created the codelist?*	Jessica Wilkins, Chloe Gao, Karina Allen, Ulrike Schmidt, Alex Dregan
		c. Date finalised	*When was the codelist finalised?*	June 2024
		d. Target data source	*What data is the codelist designed to be used with?*	Clinical Practice Research Datalink. (2025). CPRD Aurum June 2025 (Version 2025.06.001) [Data set]. Medicines and Healthcare products Regulatory Agency. https://doi.org/10.48329/a94w-e055

		e. Terminology	*What is the terminology? (e.g., SNOMED, ICD)*	MedCodeID and Term
	**Define a clinical concept**			
Define	1	a. Concept	*What is the clinical concept (e.g., the disease, drug, test result, etc…) of interest?*	Referrals to eating disorder clinics or treatment via primary care, for any eating disorder diagnosis. This may also include referrals for assessment, treatment or to clinic.
		b. Timeframe	*Should the codelist capture new, current, and/or previous events?*	New, current, and/or previous events
		c. Accuracy	*Should the codelist capture probable or definite codes?*	Probable and/or definite codes
		d. Setting	*What is the (health care) setting (e.g., primary care, hospital care)?*	Primary care
**Identify and evaluate existing codelists**				
Search	2	a. Sources searched	*Which sources were searched (e.g., internet search, codelist repositories)?*	We used this codelist repository: https://clinicalcodes.rss.mhs.man.ac.uk/

		b. Existing codelists found	*Which suitable codelists did you find?*	We did not find any suitable clinical codelists. Other codelists for eating disorder diagnoses included some referral codes in their lists e.g. https://clinicalcodes.rss.mhs.man.ac.uk/medcodes/article/78/codelist/res38-eating-disorder/
Verify	3	a. Verified by others	*Which information is available to verify the quality of suitable codelists?*	The codelist will be published in the codelist repository: https://clinicalcodes.rss.mhs.man.ac.uk/ and also be available via supplementary materials to be reviewed and replicated by others.
		b. Verified by yourself	*Which checks did you conduct to verify the quality of suitable codelists?*	Members of our research term include clinicians who work in eating disorders including a psychiatrist, clinical psychologist and counselling psychologist all of whom reviewed the codelist who used our clinical knowledge to develop search terms. Drafts of the codelist were then shared with a primary care specliast for futher verification.
Reference	4	a. Existing codelists used	*Are you making use of any existing codelists? If yes, reference these, and specify how they are being used.*	We used existing codelists created for eating disorder diagnoses (e.g. https://clinicalcodes.rss.mhs.man.ac.uk/medcodes/article/78/codelist/res38-eating-disorder/) and extracted codes relating to referrals for treatment, assessment or to clinic.
	**Create a new codelist**			
Prepare	5	a. Synonyms	*What are synonyms and related words for the clinical concept (e.g., different names for a disease/drug) and how did you identify these (e.g., source of clinical knowledge)?*	Synonyms and related words to capture eating disorder referrals include:<br>• “referral to eating disorders”<br>• “eating disorder clinic”<br>• “eating disorder treatment”<br>• “eating disorder assessment”<br>• “eating disorder management”<br>• “eating disorder*”<br><br>These synonyms were identified through close consultation with an experienced study team:<br>• Professor Ulrike Schmidt - Consultant Psychiatrist and Professor of Eating Disorders. Director of the Centre for Research on Eating and Weight Disorders at King's College London

				• Dr. Karina Allen - Consultant Clinical Psychologist in the outpatient adult eating disorder service at South London and Maudsley NHS Foundation Trust<br>• Ms. Jessica Wilkins - PhD student and HCPC registered counselling psychologist at King's College London
		b. Exceptions	*What should not be included in the codelist?*	Any codes related to eating disorder symptoms or diagnosis as these were included in a separate eating disorder diagnosis code list (also available in supplementary materials).
Create	6	a. Method used	*Which method (e.g., a script, a tool) did you use to create the draft codelist?*	We exported search results from the Clinical Practice Research Datalink Aurum code browser into an excel file. We also used a script in R to search for medical code terms in the extracted dataset from Aurum to triangulate with search results from the code browser and from existing codelists. Duplicates, identified by medcodeid (not terms) were deleted.
		b. Search terms	*Which search terms, and if applicable, exclusion terms did you use?*	CPRD aurum code browser<br>Search terms used:<br>• "eating disorder*"<br>• "referral to eating*"<br>• "*eating disorder clinic*"<br>• "eating disorder management*"<br>• "*eating disorder assessment*"
		c. Hierarchy used to extend search	*Did you use a dictionary hierarchy (e.g., ICD-10 chapters, SNOMED-CT concepts) to modify your search? If yes, specify.*	No

		d. Decisions made while iterating	*Which decisions did you make while iteratively refining the draft codelist?*	Diagnostic terms without reference to referral were eliminated.
		e. (Optional) Categories	*Did you specify subcategories within the codelist? If yes, specify.*	No
	**Review, finalise and publish**			
<u>Review</u>	7	a. Reviewers	*Who reviewed the codelist and what expertise did reviewers have?*	This codelist was verified by the following individuals:<br>• Dr. Jamie Scuffell –Population health epidemiologist and General Practitioner<br>• Professor Ulrike Schmidt - Consultant Psychiatrist and Professor of Eating Disorders. Director of the Centre for Research on Eating and Weight Disorders at King's College London<br>• Dr. Karina Allen - Consultant Clinical Psychologist in the outpatient adult eating disorder service at South London and Maudsley NHS Foundation Trust<br>• Ms. Jessica Wilkins - PhD student and HCPC registered counselling psychologist at King's College London
		b. Scope of review	*What was reviewed (Just the draft codelist or also the method, terms, etc..)?*	The draft codelist was reviewed by Dr. Scuffell, Professor Schmidt, Dr. Allen, and Ms. Wilkins. Ms. Wilkins, Professor Schmidt, and Dr. Allen reviewed methods and terms as well.
		c. Evidence of review	*Where is the review process documented?*	The review process was documented through email exchanges and in research meeting minutes.
<u>Checks</u>	8	a. Internal checks	*What method(s) were used for internal checks, if*	A search was run on the CPRD database to identify eating disorder referral codes which existed in our data set. This list was then checked against our developed codelists and additional codes were added if they appeared in the dataset.

			any, and what are the findings?	
		b. External checks	*What method(s) were used for external checks, if any, and what are the findings?*	Codelists were submitted, along with the study protocol application, for review by CPRD Research Ethics Committee.
Publish	9	a. Codelist published	*Where is the codelist published?*	The codelist will be published in the codelist repository: https://clinicalcodes.rss.mhs.man.ac.uk/
		b. Resources published	*Where are the resources used to create the codelist (e.g., scripts, list of terms)?*	We will detail our use of the Clinical Practice Research Datalink Aurum code browser and our use of an existing code list when we publish our codelist in the codelist repository, and highlight these resources in our resulting manuscripts.

b. Resources published	*Where are the resources used to create the codelist (e.g., scripts, list of terms)?*	We will detail our use of the Clinical Practice Research Datalink Aurum code browser and our use of an existing code list when we publish our codelist in the codelist repository, and highlight these resources in our resulting manuscripts.

Note. An Explanation and Elaboration article discusses each checklist item and gives methodological background and published examples of transparent reporting. Matthewman J, Andresen K, Suffel A, Lin LY, Schultze A, Tazare J, Bhaskaran K, Williamson E, Costello R, Quint J, Strongman H. Checklist and guidance on creating codelists for routinely collected health data research. NIHR Open Res. 2024 Sep 18;4:20. doi: 10.3310/nihropenres.13550.2. PMID: 39345273; PMCID: PMC11437289.The Codelist checklist is best used in conjunction with this article.

Supplementary Materials 2.5 Consultation Codelists

Id	Description	category
1710	Acute visit	face to face
1801	Attendance	face to face
1561	Baby Clinic	face to face
229	Booked Appointment	face to face
1865	Branch Surgery	face to face
364	Clinic NHS	face to face
397	Community Clinic	face to face
2324	Community health clinic	face to face
421	Consultation	face to face
18347	Consultation via video conference	telephone/virtual
32234	Contact method: Acute visit	face to face
4840	Contact method: Branch Surgery	face to face
115108	Contact method: By Appointment	face to face
31854	Contact method: Clinic	face to face
31967	Contact method: Community Clinic	face to face
31844	Contact method: Community Place of consultation	face to face
204690	Contact method: Duty Doctor Urgent Appointment	face to face
32000	Contact method: Emergency Appointment	face to face
31821	Contact method: Emergency Consultation	face to face
426	Contact method: G.P.Surgery	face to face
208309	Contact method: G.P.Surgery	face to face
4839	Contact method: GP Practice	face to face
31907	Contact method: Gp Triage Consultation	face to face
31877	Contact method: H Docs Advice	face to face
32066	Contact method: Main Surgery	face to face
115109	Contact method: Nurse Triage	face to face
32067	Contact method: Nurse Triage Consultation	face to face
208308	Contact method: On Call Pm	face to face
31960	Contact method: Out Of Hrs Telephone	telephone/virtual
32077	Contact method: Seen in GPs surgery	face to face
55912	Contact method: Surgery Attendance	face to face
32262	Contact method: Surgery consultation	face to face
7303	Contact method: Telephone Advice	telephone/virtual
32001	Contact method: Telephone call to a patient	telephone/virtual
32163	Contact method: Telephone encounter	telephone/virtual

7304	Contact method: Triage	face to face
31805	Contact method: Triage Telephone Call	telephone/virtual
31900	Contact method: Urgent Appointment	face to face
32301	Contact method: Urgent Consultation	face to face
7361	Daytime Visits patients home	face to face
7395	Diabetic Clinic	face to face
2527	Duty Doctor Telephone	telephone/virtual
5037	Duty Doctor Urgent Appointment	face to face
5040	Duty Telephone Appt	telephone/virtual
47488	E-mail Dialogue	telephone/virtual
62015	Email Clinical	telephone/virtual
608	Email Consultation	telephone/virtual
21613	Email Encounter	telephone/virtual
5083	Emergency Appointment	face to face
2579	Emergency consultation	face to face
638	Emergency Doctor	face to face
2580	Emergency Gp Surgery	face to face
5093	Emergency Nurse Clinic	face to face
5097	Emergency Surgery	face to face
5136	Extra Appointment	face to face
5149	Face to face consultation	face to face
7609	Follow-up/routine visit	face to face
745	G P Consultation	face to face
7629	G P Ordinary Surgery	face to face
2711	G.P Surgery (Pm)	face to face
751	G.P. Evening Surgery	face to face
2719	G.P. Morning Surgery	face to face
5252	G.P.Surgery Urgent Consultation	face to face
58	GP Practice	face to face
236755	Home	face to face
7883	Home of Patient	face to face
212572	Home Visit	face to face
984	Home Visit - In Surgery Hours	face to face
5464	Home visit note	face to face
3271	Main Surgery	face to face
32429	Night Visit	face to face
3441	Night visit , practice	face to face
3430	Night Visit - patient's home	face to face
3438	Night Visit patients home	face to face

8325	Night visit, Deputising service	face to face
3440	Night visit, Local rota	face to face
8348	Normal Home Visit (08:00 - 11:00)	face to face
3499	Nurse Assessment Clinic	face to face
3503	Nurse Minor Illness Clinic	face to face
3504	Nurse Practitioner	face to face
3505	Nurse Practitioner Surgery	face to face
3506	Nurse Practitioner Telephone Advice	telephone/virtual
3507	Nurse Run Family Planning Clinic	face to face
3508	Nurse Sexual Health Appointment	face to face
3509	Nurse Surgery	face to face
3510	Nurse Surgery Triage	face to face
5946	Nurse telephone triage	telephone/virtual
3511	Nurse Triage	face to face
3512	Nurse Triage Clinic	face to face
3513	Nurse Triage Consultation	face to face
3514	Nurse Visit	face to face
5952	Nurse's Treatment Room Clinic	face to face
8397	Nurses Room	face to face
3516	Nurses' Flu Clinic	face to face
8400	Nursing Home	face to face
8703	Nursing home visit note	face to face
6006	Open Access Surgery	face to face
8446	OPEN DOOR SURGERY	face to face
6008	Open Surgery	face to face
6065	Out of Hours	face to face
3619	Out Of Hours Advice	face to face
3620	Out Of Hours Attendance	face to face
3621	Out Of Hours Calls	face to face
3622	Out Of Hours Centre	face to face
8799	Out of hours consultation at surgery	face to face
3626	Out Of Hours Gp Service	face to face
3627	Out Of Hours Gp Visit	face to face
3628	Out Of Hours Home Visit	face to face
3631	Out Of Hours Night Visit	face to face
3633	Out Of Hours Prim Care Centre	face to face
3636	Out Of Hours Telephone Advice	telephone/virtual
3637	Out Of Hours Telephone Call	telephone/virtual
3638	Out Of Hours Telephone Consultation	telephone/virtual

3639	Out Of Hours Telephone Contact/Advice	telephone/virtual
8812	Out Of Hours Visit	face to face
6087	Out of hours, Practice	face to face
3640	Out Of Hours-Telephone Advice	telephone/virtual
6140	P.Nurse Clinic	face to face
9824	Patient encounter data NOS	face to face
6207	Phone	telephone/virtual
9907	Practice Nurse	face to face
6236	Practice Nurse Clinic	face to face
3788	Practice Nurse Surgery	face to face
9927	Primary Care Centre	face to face
8933	Primary care organisation	face to face
9060	Residential Home	face to face
9061	Residential home visit note	face to face
6426	Same Day Appointment	face to face
4020	Same Day Clinic	face to face
6440	Saturday Morning Surgery	face to face
9203	Seen by Practice Nurse	face to face
10149	Seen in GP's surgery	face to face
6513	Seen in Health Centre	face to face
9217	Seen in Nurses Surgery	face to face
187348	Seen in own home	face to face
6519	Seen out of hours	face to face
4226	Surgery	face to face
4215	Surgery Attendance	face to face
4217	Surgery Clinic	face to face
4221	Surgery consultation	face to face
6652	Surgery Emergency	face to face
9361	Surgery or Clinic	face to face
4281	Telephone	telephone/virtual
20536	Telephone & Sub. Visit	telephone/virtual
4271	Telephone (Triage)	telephone/virtual
208423	Telephone (Visits)	telephone/virtual
14781	Telephone 1st	telephone/virtual
14173	Telephone 1st - Successful	telephone/virtual
14775	Telephone 2nd	telephone/virtual
9412	Telephone 2nd - Successful	telephone/virtual
54745	Telephone 3rd	telephone/virtual
55223	Telephone 3rd - Successful	telephone/virtual

208323	Telephone 4th	telephone/virtual
114642	Telephone 4th - Successful	telephone/virtual
4283	Telephone Advice	telephone/virtual
4286	Telephone Advice By G.P	telephone/virtual
14230	Telephone Advice By Nhs Direct	telephone/virtual
49506	Telephone Advice By Nurse	telephone/virtual
4288	Telephone Appt	telephone/virtual
6728	Telephone call from a patient	telephone/virtual
9417	Telephone call from relative/carer	telephone/virtual
6730	Telephone call to a patient	telephone/virtual
6731	Telephone call to relative/carer	telephone/virtual
6736	Telephone call with Patient	telephone/virtual
4293	Telephone Consultation	telephone/virtual
4298	Telephone encounter	telephone/virtual
6712	Telephone Surgery	telephone/virtual
52083	Telephone Triage	telephone/virtual
9433	Telephone Triage By Doctor	telephone/virtual
6751	Telephone triage encounter	telephone/virtual
62529	Telephone with patient/carer	telephone/virtual
53039	Telephone, Acute visit	telephone/virtual
52665	Telephone, Follow-up/routine visit	telephone/virtual
204223	Telephone, Health Visiting	telephone/virtual
21411	Telephone, Home Visit	telephone/virtual
90386	Telephone, Night Visit	telephone/virtual
73235	Telephone, Night visit, Deputising service	telephone/virtual
21527	Telephone, Night visit, Local rota	telephone/virtual
98289	Telephone, Nursing Home Visit	telephone/virtual
101218	Telephone, Residential Home Visit	telephone/virtual
4309	Telephone: Nurse Triage	telephone/virtual
6820	Three Minute Surgery	face to face
4387	Treatment Room	face to face
6838	Treatment Room (Nurse)	face to face
10510	Triage	face to face
4394	Triage By Phone	telephone/virtual
9551	Unbooked Clinic	face to face
10591	Urgent Appointment	face to face
4487	Urgent Slots	face to face
6923	Urgent Surgery	face to face
4506	visit	face to face

10627	Visit-Home	face to face
6963	Walk-In Surgery	face to face
4545	Weekday Surgery	face to face

Job Category Codelist

jobcatid	Description
4	General Medical Practitioner
5	Salaried General Practitioner
8	Community Practitioner
9	Community Nurse
11	Dietitian
14	Health Care Support Worker
15	Associate Practitioner - General Practitioner
17	Phlebotomist
24	GP Registrar
27	Specialist Nurse Practitioner
31	Sessional GP
33	Nurse Consultant
36	Healthcare Assistant
47	Staff Nurse
55	Associate Practitioner - Nurse
73	Practitioner
87	Associate Practitioner
91	Specialist Registrar
116	House Officer - Post Registration
118	Dietitian Specialist Practitioner
119	Trust Grade Doctor - SHO level
126	Trust Grade Doctor - Career Grade level
181	Locum GP
183	Assistant GP
186	Health Visitor
189	Physician Assistant
190	Deputising Doctor
197	Trust Grade Doctor - House Officer level
251	Physician Associates
252	Dietitian Advanced Practitioner

Consultation Medcodeid List

medcodeid	Term
282615013	Emergency treatment
282617017	Emergency treatment NOS
283520011	Emergency hospital admission
283524019	Emergency psychiatric admission under Mental Health Act 1983 (England and Wales)
285368015	Emergency appointment
4717611000006110	Emergency care
6445511000006110	Emergency treatment assessment
6445551000006110	Emergency treatment management
6514941000006110	Emergency mental health assessment
6517771000006110	Emergency mental health assessment - 24 hour intensive
6517781000006110	Emergency mental health assessment - 24 hour not intensive
285194015	Seen in hospital casualty
667121000000119	Seen in accident and emergency department
1488307015	Accident & emergency
285266019	Seen in emergency clinic
841501000006118	A&E attendance
8013251000006110	Out of hours report
1672471000006110	Clinic
849441000006118	Follow-up
388001000027116	Out of Hours
63451000000117	Night visit note
63461000000119	Night visit practice note
8052771000006110	Walk-in Centre Event
62201000000114	Acute visit note
1809171000006110	Routine consultation
2019541000006110	Routine appointment
8049651000006110	Out of Hours Event

8276311000006110	Out of hours service
19773013	Consultation
285405016	New patient consultation
418934011	Follow-up consultation
34361000000116	Weekend consultation at surgery
34111000000115	Out of hours consultation at surgery
1664351000006110	Consultation purpose type - General
1664341000006110	Consultation purpose type - Investigation
1664331000006110	Consultation purpose type - Treatment
1664321000006110	Consultation purpose type - Review
1771751000006110	Consultation for minor illness
1809161000006110	Urgent consultation
1809181000006110	Emergency consultation
1809191000006110	Extended hours consultation
1823481000006110	Acrimonious doctor/patient consultation
1849981000006110	Follow up telephone consultation
2322391000000110	Joint consultation
7507561000006110	Consultation for treatment
8105151000006110	Face to face consultation
8238881000006110	Consultation with patient
265637019	Patient review
285185014	Seen in GP's surgery
283022014	Had a chat to patient
1780266016	Mental health review
282965018	Patient given advice
285238010	Seen in general medical clinic
285279011	Seen by general practitioner
285281013	Seen by practice nurse
285292014	Seen by counsellor
405038017	Seen in own home

285312012	Seen by co-operative doctor
285458014	Visit out of hours
301501000000119	Seen by health care assistant
459247016	Seen by triage nurse
1672851000006110	Face to face consultation
63311000000113	Repeat issue note
451201014	Weight monitoring
282730013	Medication requested
458905016	Medication review done
1780456010	Telephone triage encounter
2286871000000110	NHS 111 report received
2534091015	Depression interim review
712411000006117	Medication review
272571000000119	Seen in out of hours centre
497391000000118	Seen in walk in centre
2160114019	Medication review done by doctor
14176391000006100	Referral to weight management service
285327012	Telephone encounter
1480626017	Telephone follow-up
62141000000119	Telephone call to a patient
62151000000116	Telephone consultation
63381000000118	Telephone call from a patient
8012271000006110	Telephone consultation
8217951000006110	Telephone contact only
1539091000006110	First telephone or telemedicine consultation
1539101000006110	Follow-up telephone or telemedicine consultation
1778971000006110	Consultation via telemedicine web camera
1778991000006110	Consultation via SMS text message
1779001000006110	Other consultation medium used
2687891000006110	Telephone call by physician to patient or for consultation

3748121000006110	Psychiatric telephone consultation or therapy with patient
7353351000006110	Telemedicine consultation with patient
8361131000006110	Consultation via multimedia
8442071000006110	eConsultation via online application
12000781000006100	Telepractice consultation
14393881000006100	Consultation via video consultation
8105161000006110	Remote consultation
8105171000006110	Remote verbal consultation
8105181000006110	Consultation by telephone
1849991000006110	First telephone consultation
494431000000117	SMS text message received from patient
1995221000006110	Email encounter
11990111000006100	Online triage encounter
457040019	Patient given telephone advice out of hours
283763012	Planned telephone contact
1773301000006110	E-mail consultation
61881000000113	Message from patient

Supplementary Materials

Table SM3.

Missing data by case status

Variable	Controls: N missing (%)	Cases: N missing (%)
Ethnicity	9,682 (6.2%)	1,946 (4.2%)
BMI	46,489 (32.0%)	7,258 (15.6%)
Smoking Status	9, 751 (6.7%)	2,161 (4.7%)

Note. BMI = Body Mass Index

Supplementary Materials 4

Table 4.1

Sensitivity analysis of the association between eating disorder case status and primary care consultation rates, by smoking data inclusion

Model	N	Smoking data included	IRR (case vs. control)	95% CI	% change vs. primary model
Model A	179,847	Smoking categories 1 & 2 only	1.962	1.941-1.984	Reference
Model B	191,759	Smoking Categories 1, 2 & 3	1.955	1.934-1.976	-0.39%

Note. IRRs were estimated using fixed-effects negative binomial regression with matched-set fixed effects (age, sex, and practice). Model A excluded individuals with missing smoking status, while Model B additionally included individuals coded as smoking status category 3. Estimates were highly consistent across models, indicating minimal impact of missing smoking data on the association between eating disorder case status and consultation rates.

Table 4.2

Sensitivity analysis for fixed effects negative binomial regression models with and without BMI

Diagnosis	IRR (no BMI)	IRR (with BMI)	% change
AN	1.78	1.79	+0.39
BN	1.88	1.77	-5.70
OSFED	1.83	1.81	-0.97
BED	2.08	1.77	-14.65
ARFID	2.45	2.49	+1.68
PICA	2.25	2.18	-3.24
ED-unspecified	2.04	2.01	-1.66
Mixed ED	2.32	2.30	-0.76

Note. IRRs were obtained from fixed-effects negative binomial models with matched-set fixed effects (age, sex, practice). The BMI-adjusted model additionally included continuous BMI. Percentage change reflects attenuation of diagnosis-specific IRRs after BMI adjustment. Reference group is matched healthy controls. The model without BMI included 161,698 individuals; the BMI-adjusted model included 123,204 individuals.

Table SM 5.1

Adjusted predicted probability of ever being referred, by ethnicity and BMI category

Ethnicity	BMI Category	Predicted Probability	95% CI
White (ref)	Normal (ref)	0.128	0.107-0.153
White	Underweight	0.210	0.177-0.247
White	Overweight	0.094	0.077-0.114
White	Obese I	0.104	0.085-0.127
White	Obese II+	0.134	0.111-0.162
Asian	Normal	0.103	0.081-0.130
Asian	Underweight	0.181	0.140-0.230
Asian	Overweight	0.060	0.041-0.088
Asian	Obese I	0.082	0.052-0.126
Asian	Obese II+	0.091	0.057-0.142

Black	Normal	0.077	0.053-0.108
Black	Underweight	0.165	0.106-0.248
Black	Overweight	0.083	0.054-0.128
Black	Obese I	0.040	0.019-0.081
Black	Obese II+	0.153	0.106-0.216
Mixed	Normal	0.151	0.112-0.200
Mixed	Underweight	0.267	0.181-0.376
Mixed	Overweight	0.112	0.071-0.173
Mixed	Obese I	0.125	0.072-0.210
Mixed	Obese II+	0.152	0.090-0.246
Other	Normal	0.090	0.047-0.167
Other	Underweight	0.241	0.108-0.456
Other	Overweight	0.087	0.025-0.257
Other	Obese I	0.158	0.019-0.648
Other	Obese II+	0.142	0.014-0.662
Unknown	Normal	0.121	0.095-0.154
Unknown	Underweight	0.191	0.143-0.249
Unknown	Overweight	0.107	0.071-0.156
Unknown	Obese I	0.122	0.072-0.199
Unknown	Obese II+	0.153	0.099-0.229

Note. Predicted probabilities were estimated from a logistic regression model including an ethnicity × BMI interaction, adjusted for age, sex, smoking status, IMD quintile, and diagnosis category. Estimates are marginal means averaged over covariate distributions and are presented on the probability scale.

Table SM 5.2

Risk differences in ED referral by ethnicity within Body Mass Index (BMI) category

BMI category	Contrast	Risk difference (pp)	95% CI	p-value
Normal	Asian vs White	−2.5	−5.1 to −0.1	0.064
Normal	Black vs White	−5.1	−8.8 to -1.5	<0.001
Normal	Mixed vs White	+2.3	−2.9 to +7.6	0.810
Normal	Other vs White	−3.8	−11.9 to +4.4	0.773
Underweight	Asian vs White	−2.9	−8.2 to +2.3	0.611
Underweight	Black vs White	−4.5	−17.1 to +3.4	0.643
Underweight	Mixed vs White	-2.1	-12.2 to +8.0	0.991
Underweight	Other vs White	-3.9	-24.3 to 16.6	0.994
Overweight	Asian vs White	−3.3	-6.5 to -0.2	0.034
Overweight	Black vs White	-1.1	-6.0 to 3.9	0.991
Overweight	Mixed vs White	+1.8	-5.0 to 8.6	0.974
Overweight	Other vs White	-0.7	-15.5 to 14.1	1.000
Obese I	Asian vs White	-2.2	-7.3 to 2.9	0.815
Obese I	Black vs White	−6.4	-11.0 to -1.8	<0.001
Obese I	Mixed vs White	+2.2	-7.3 to 11.6	0.987
Obese I	Other vs White	+5.4	-38.7 to 49.5	0.999
Obese II+	Asian vs White	-4.4	-10.3 to 1.5	0.269
Obese II+	Black vs White	+1.8	-5.4 to 9.0	0.979
Obese II+	Mixed vs White	+1.8	-8.9 to 12.4	0.997
Obese II+	Other vs White	+0.8	-42.8 to 44.3	1.000

Note. Risk differences (percentage points) were estimated from marginal predicted probabilities derived from a logistic regression model including an ethnicity × BMI interaction, adjusted for age, sex, smoking status, IMD quintile, and diagnosis category. Estimates are averaged over covariate

distributions and presented on the probability scale. P-values are Tukey-adjusted for multiple comparisons within each BMI category.

Table SM 5.3

Risk differences in ED referral by Body Mass Index (BMI) category within ethnicity

Ethnicity	Contrast	Risk difference (pp)	95% CI	p-value*
White	Underweight vs Normal	+8.2	+5.9 to +12.5	<0.001
White	Overweight vs Normal	−3.4	−4.8 to −2.0	<0.001
White	Obese I vs Normal	-2.4	-4.1 to -0.8	<0.001
White	Obese II+ vs Normal	+0.6	-1.0 to 2.3	0.805
Asian	Underweight vs Normal	+7.8	+2.3 to +13.3	0.001
Asian	Overweight vs Normal	−4.2	−8.0 to −0.5	0.018
Asian	Obese I vs Normal	-2.1	-7.3 to 3.1	0.799
Asian	Obese II vs Normal	-1.2	-7.1 to 4.6	0.979
Black	Underweight vs Normal	+8.8	-1.0 to 18.6	0.099
Black	Overweight vs Normal	+0.7	-4.9 to 6.3	0.997
Black	Obese I vs Normal	-3.7	-8.8 to 1.4	0.279
Black	Obese II+ vs Normal	+7.6	0.00 to 15.3	0.052
Mixed	Underweight vs Normal	+3.8	-7.0 to 14.5	0.873
Mixed	Overweight vs Normal	-3.9	-12.1 to 4.3	0.689
Mixed	Obese I vs Normal	-2.6	-12.8 to 7.6	0.958
Mixed	Obese II vs Normal	+0.1	-11.2 to 11.4	1.000
Other	Underweight vs Normal	+8.1	-12.9-29.0	0.833
Other	Overweight vs Normal	-0.3	-16.4 to 15.7	1.000
Other	Obese I vs Normal	+6.7	-36.1 to 49.6	0.993
Other	Obese II+ vs Normal	+5.2	-37.3 to 47.6	0.997
Unknown	Underweight vs Normal	+7.0	0.0 to 13.9	0.050
Unknown	Overweight vs Normal	-1.5	-7.6 to 4.6	0.966
Unknown	Obese I vs Normal	+0.1	-8.6 to 8.8	1.000

Ethnicity	Contrast	Risk difference (pp)	95% CI	p-value*
Unknown	Obese II vs Normal	+3.2	-5.6 to 12.0	0.859

Note. Risk differences (percentage points) were derived from marginal predicted probabilities estimated from a logistic regression model including an ethnicity × BMI interaction and adjusted for age, sex, smoking status, IMD quintile, and diagnosis category. Standard errors and 95% confidence intervals were obtained using the delta method. P-values were Tukey-adjusted for multiple comparisons within ethnicity.

Table SM 5.4

Adjusted probability of eating disorder referral by UK region

Region	Adjusted referral probability	95% CI	p-value
London (ref)	0.130	0.112-0.151	--
North East	0.043	0.034-0.055	<0.001
North West	0.168	0.146-0.193	<0.001
Yorkshire and The Humber	0.092	0.075-0.112	<0.001
East Midlands	0.106	0.086-0.130	0.012
West Midlands	0.126	0.108-0.147	0.505
East of England	0.136	0.113-0.163	0.570
South East	0.160	0.139-0.183	<0.001
South West	0.078	0.066-0.092	<0.001
Northern Ireland	0.113	0.057-0.211	0.662

Note. Estimates are predicted probabilities derived from a logistic regression model adjusted for age, sex, smoking status, Index of Multiple Deprivation (IMD) quintile, and eating disorder diagnosis. London was specified as the reference region. 95% confidence intervals (CIs) are shown. P-values correspond to comparisons with the reference region. The overall association between region and referral was statistically significant (Wald test $p < 0.001$).
