# Supplementary material for: Primary care use among adults with eating disorders in England: a population-based cohort study using electronic health records
Source: BMJ Open. 2026 Jun 24;16(6):e119143. doi: 10.1136/bmjopen-2026-119143 (PMC13295994; doi:10.1136/bmjopen-2026-119143)
Supplement: Supplementary file 2 [file bmjopen-2026-119143.reviewer_comments.pdf]

PEER REVIEW HISTORY

BMJ Open publishes all reviews undertaken for accepted manuscripts. Reviewers are asked to complete a checklist review form and are provided with free text boxes to elaborate on their assessment. These free text comments are reproduced below.

ARTICLE DETAILS

Title (Provisional)

Primary care use among adults with eating disorders in England: a population-based cohort study using electronic health records

Authors

Wilkins, Jessica; Gallagher, Lucy; Allen, Karina; Dregan, Alexandru; Gao, Chloe; Scuffell, Jamie; Schmidt, Ulrike

---

VERSION 1 - REVIEW

**Reviewer**	**1**
**Name**	**Dack, Kyle**
**Affiliation**	**University of Bristol, MRC Integrative Epidemiology Unit**
**Date**	**23-Mar-2026**
**COI**	**None**

Statistical review summary:

The methods and results of this study are reported clearly and adhere to STROBE recommendations. The statistical analysis is reasonable and the data is interpreted suitably, and I have no major concerns. My comments below are minor points of clarification or queries, the most substantial relating to whether differences in clinical practice could have occurred over the long study dates (13 years).

1. Checklist - This study uses routinely collected data (EHR), rather than data specific to a study or cohort. As such, while STROBE is useful and relevant, I suggest considering to attach the RECORD extension as well, which is specifically designed for studies using this form of data: https://resources.equator-network.org/reporting-guidelines/record/

Abstract:

2. The abstract reports all essential methodological elements, and the statistics are clear - I do not have comments or suggestions.

Introduction:

3. Aims 1 and 2: It would be helpful to clarify that these aims are concerned with contact rates in the two years prior to diagnosis/referral, as the current phrasing could be misinterpreted as comparing rates in those already diagnosed. Or by rephrasing “individuals with ED” to individuals with undiagnosed ED, or similar.

Methods:

4. Data sources - EHR appears to have been autocorrected to HER.

5. Study population - “The index date was defined as the first date of ED diagnosis or referral during the follow-up period.” I think these are in order of preference, i.e. diagnosis date was prioritized, but if it was missing then referral date was used, but I suggest to confirm this in text.

6. “Controls were required to be ED free during the study period” - how was this determined?

7. “[controls] were allocated an index date defined as the date of primary care contact occurring at a similar time point to the matched case’s index date, which reduced the likelihood of selecting controls with infrequent primary care use.”

I suggest adding the specific time window used to match to the case’s index date.

I could not follow how this would reduce the selection of controls with infrequent primary care use, or why that would be desirable? I can see it being useful as a means of minimizing confounding by year, but could more be added to elaborate?

8. Modelling strategy: Overall the modelling approach looks reasonable, and I agree with the decision to omit BMI, because it may in part be a consequence of ED and therefore a mediator rather than confounder, which could split the association of interest. Confounding was not directly mentioned in the methods - I imagine it is the rationale for the matched variables and adjustment for smoking, but I recommend explicitly confirming if this was the case, to reassure readers confounding bias was considered.

Results

9. RQ 1: It may be helpful to remind readers of the study context, e.g. “Individuals with an ED had a higher mean number of total primary care contacts than controls [over the two years prior to the index date] (33.1 vs 20.2)”

10. “in adjusted negative binomial regression analyses” - I suggest reminding readers what was adjusted for.

11. Table 3, 4, 5: While it is in the text, for readers who skip to the table, I suggest adding the model type, and covariates.

12. “Compared with White individuals, the excess consultation rate among cases was significantly greater for Asian (IRR 1.12)” I could not follow how this would be interpreted as

excess consultations, as this was not previously mentioned in the paper - could the authors please clarify?

Discussion

13. The study date range of 2010-2023 is quite wide. While it strengthens the statistical power, it may also risk introducing temporal heterogeneity. It is possible that there are temporal trends such as changes in clinical practice, EHR data quality or recording/coding practices, or changes in awareness of ED which lead to changes in patient behaviour (e.g. more willing to request referrals).

It would be interesting in the discussion to hear the authors thoughts on this possibility, and whether it impacts the results. It could also be informative to describe the distribution of cases, e.g. a histogram of index dates.

Reviewer 2

Name Jenkins, Paul

Affiliation University of Reading, School of Psychology and Clinical Language Sciences

Date 04-May-2026

COI None

Thank you for the opportunity to review this paper, which looks at frequency of contact in primary care by adults with a diagnosis of an ED. The manuscript is generally clear, presentation of data (including in SM) seems thorough, and the findings are important. I have some comments the authors might wish to consider.

I think the use of "UK" is potentially misleading (given that data from Wales and Scotland are not included), and I invite the authors to think about this, mindful that this could become complicated. Population figures might also need to be adjusted should this language be changed.

Why was an upper limit of 65y used? Similarly, the timeframe of 24m could be better justified. Is it possible to say whether there are patterns within this 24m – e.g., clustering at the beginning, or end?

I was a little unclear on how the codelist was used (mindful that variations have been used before). For instance, if "anorexia" was noted, how was this distinguished from "anorexia nervosa"? A little more detail is important for readers to have confidence that the psychiatric conditions of EDs are being considered, rather than 'organic' or non-specific issues (e.g., loss of appetite).

What was the cutoff (e.g., in mmol/L) for hypokalaemia used? (I think 3.5 is mentioned in the SM, which could be stated in the main text.) Given that this did not really feature in the

analyses, some of the detail (e.g., in Table 1) might be removed, perhaps only keeping reference to the fact that this was planned as a covariate, but not used (p. 13).

Why were analyses adjusted for smoking status (only)?

A potential limitation which could be noted (e.g., p. 26) is that language proficiency (for minoritised groups, but perhaps more widely) was not considered / available, and this could be related to uptake / access.

Statistics 'demonstrating' equivalence between the two groups might be useful in Table 2.

A citation is needed (p. 23, ll26–27) regarding "evidence that Black individuals… often have higher BMI and more severe eating-related concerns" than community samples.

The findings on referral rates are interesting (albeit disappointing). Are the authors able to comment on whether this categorisation covers 'attempted' referrals, and/or whether these were 'accepted' by the EDS? This might then affect GP contact rates, although perhaps not in predictable directions (e.g., referral to EDS reduces GP contact as patient is under a 'specialist' but could also increase contact due to requests for medical monitoring).

There are two findings which stood out to me and which I felt were discussed in limited depth. First, the 'prevalence' of AN was notably high relative to other EDs, particularly in light of community estimates (e.g., van Eeden et al., 2021, Current Opinion in Psychiatry). Could the authors comment on why the number of such cases was so apparently high in the current study?

Relatedly, the OSFED category showed fairly high primary care use and other idiosyncrasies (e.g., wide BMI range). Could the authors comment on the types of presentations which might fall into this group, even if that is broad? Given its relative prevalence (as the third most common diagnostic label) and issues around identification (e.g., Attia & Guarda, 2024, IJEDs; Jenkins et al., 2021, BJPsych Open), further comment on this might be warranted.

Finally, this might be outside of the scope of the research, but I was curious whether contact rates varied according to ED severity as specified in the DSM, for instance. It may not be possible (e.g., p. 24, ll41–43), but it would be interesting if these data were available.

Some minor suggestions:

There is some repetition (of primary care) – e.g., in the Title, objectives

The Title could specify that this was a study of adults

There are some typographical errors (e.g., Abstract – "had diagnosis of anorexia"; "detailed by Wyatt et al.'s (26) to identify"; "studies [e.g. (26-28) and"; "These finding")

HER should be EHR (p. 7)

Some phrasing could be more specific, e.g., participants were "required to be ED free" – presumably this refers to an absence of an ED diagnosis on their records?

Pica does not need to be capitalised (several places)

Individuals with EDs are noted as having "higher comorbidities" – relative to whom? Non-ED cases? Other MH problems?

VERSION 1 - AUTHOR RESPONSE

**Reviewer #1 Dr. Kyle, Dack, University of Bristol**		
The methods and results of this study are reported clearly and adhere to STROBE recommendations. The statistical analysis is reasonable and the data is interpreted suitably, and I have no major concerns. My comments below are minor points of clarification or queries, the most substantial relating to whether differences in clinical practice could have occurred over the long study dates (13 years).	We thank you for this positive feedback.	N/A
1. Checklist - This study uses routinely collected data (EHR), rather than data specific to a study or cohort. As such, while STROBE is useful and relevant, I suggest considering to attach the RECORD extension as well, which is specifically designed for studies using this form of data: https://resources.equator-network.org/reporting-guidelines/record/	We thank you for this suggestion. We have now added the RECORD checklist as well, and this is found in the Supplementary Materials alongside the STROBE checklist.<br><br>Where necessary, we made changes in the revised manuscript to adhere to the RECORD guidelines.<br><br>We have also added a reference in the text of the revised manuscript:<br><br>*"This study was reported in accordance with the STROBE and RECORD reporting guidelines (SM 1)."*	Supplementary Materials 1<br><br><br><br><br><br><br><br>p. 7<br>Line: 12
2. The abstract reports all essential methodological elements, and the statistics are clear - I do not have comments or suggestions.	Thank you for this positive feedback.	N/A

Introduction:<br><br>3. Aims 1 and 2: It would be helpful to clarify that these aims are concerned with contact rates in the two years prior to diagnosis/referral, as the current phrasing could be misinterpreted as comparing rates in those already diagnosed. Or by rephrasing "individuals with ED" to individuals with undiagnosed ED, or similar.	Thank you for this feedback and the opportunity to clarify our description of the aims in the introduction. We have revised aims 1 and 2 according to your suggestion so they now read:<br><br>*"1. Do primary care contact rates during the 24 months preceding an ED diagnosis/referral differ between individuals with EDs compared to non-ED controls, matched by age, sex and practice location?*<br>*2. Among individuals with EDs, to what extent do contact rates during the 24 months preceding diagnosis or referral vary by ethnic background?"*	p. 6, Lines 25-27<br>p. 7, Lines: 1-2
Methods:<br><br>4. Data sources - EHR appears to have been autocorrected to HER.	Thank you for highlighting this typographical error. This has now been corrected in the manuscript.	p. 7, Line 19
5. Study population - "The index date was defined as the first date of ED diagnosis or referral during the follow-up period." I think these are in order of preference, i.e. diagnosis date was prioritized, but if it was missing then referral date was used, but I suggest to confirm this in text.	Thank you for this comment.<br><br>We have now clarified this (along with our rationale) in the Study Population section of the Methods:<br><br>*"The index date was defined as the first date of ED diagnosis during the follow-up period, or the date of referral to an ED service where no diagnosis was recorded. Individuals referred to specialist ED services without a recorded ED diagnosis in primary care were included to capture probable ED presentations where GPs may not have assigned an ED diagnostic code prior to referral."*	p. 8<br>Lines:12-16
6. "Controls were required to be ED free during the study period" - how was this determined?	We have used the ED medical codes to identify all (prevalent and incident) patients in the databases, based on the study ED codelists. Individuals with any ED diagnosis or referral code were therefore excluded from the pool of	p. 8<br>Lines: 18-20

	eligible controls. We have clarified how we determined controls in the *Study Population* sub-section of the Methods:<br><br>*"Controls were required to have no recorded ED diagnosis or referral in their CPRD record, based on the study codelists (Table 1)."*	
7. "[controls] were allocated an [defined as the date of primary care contact occurring at a similar time point to the matched case's index date, which reduced the likelihood of selecting controls with infrequent primary care use."<br><br>I suggest adding the specific time window used to match to the case's index date.<br><br>I could not follow how this would reduce the selection of controls with infrequent primary care use, or why that would be desirable? I can see it being useful as a means of minimizing confounding by year, but could more be added to elaborate?	Thank you for the opportunity to clarify our selection of the control group. Matching controls on the case index date is necessary to ensure that the control was alive and contributed data to CPRD at the time of a case definition. This is needed to ensure that the comparison is based on a concurrent at-risk population, thus eliminating the risk of immortal time bias and ensuring that both cases and controls had an equal opportunity to be captured within the care system during the same period of medical practice and data recording standards. We have added additional information to clarify how the index date for controls was defined. Changes are highlighted in red:<br><br>*"The index date was defined as the first date of ED diagnosis during the follow-up period, or the date of referral to an ED service where no diagnosis was recorded. Individuals referred to specialist ED services without a recorded ED diagnosis in primary care were included to capture probable ED presentations where GPs may not have assigned an ED diagnostic code prior to referral. GP records were extracted for the 24 continuous months prior to this index date. Each patient with an ED was exactly matched to up to 3 controls without ED on age, sex, GP practice ID, and index date. Controls were required*	p. 8<br>Lines: 12-25

	to have no recorded ED diagnosis or referral in their CPRD record, based on the study codelists (Table 1). They were assigned the same index date as their matched case. To ensure this was a valid time point for the control, we required the cases' index date fell within the control's period of active registration at the same practice. This ensured that the controls were under clinical observation during the same time as their matched case, reducing the likelihood of selecting controls with infrequent primary care use. It also helped to account for temporal changes in clinical practice, coding behaviour, and EHR recording over the study period."	
8. Modelling strategy: Overall the modelling approach looks reasonable, and I agree with the decision to omit BMI, because it may in part be a consequence of ED and therefore a mediator rather than confounder, which could split the association of interest. Confounding was not directly mentioned in the methods - I imagine it is the rationale for the matched variables and adjustment for smoking, but I recommend explicitly confirming if this was the case, to reassure readers confounding bias was considered.	Thank you for your comment and the opportunity to clarify how we addressed confounding bias in our analyses.<br><br>We have revised the Methods section to explicitly state which variables were controlled through matching and which were included as covariates in the regression models.<br><br>*"Models compared individuals within matched case-control sets. Age, sex, GP practice and index date were therefore controlled by design through matching and by inclusion of matched-set fixed effects. Smoking status was included as a covariate because it was not part of the matching strategy and may be associated with healthcare use (30)."*	p. 13<br>Lines:13-17
9. RQ 1: It may be helpful to remind readers of the study context, e.g. "Individuals with an ED had a higher mean number of	Thank you for this comment. We have updated the manuscript (change in red):	p. 17<br>Lines: 12-14

total primary care contacts than controls [over the two years prior to the index date] (33.1 vs 20.2)"	"Individuals with an ED had a higher mean number of total primary care contacts than controls over the two years prior to the index date (33.1 vs 20.2), including face-to-face contacts (14.9 vs 10.1) and telephone or virtual contacts (14.3 vs 7.4)."	
10. "in adjusted negative binomial regression analyses" - I suggest reminding readers what was adjusted for.	Thank you for this comment, we have integrated your suggestion to remind readers of what was adjusted for in this part of our analysis (changes in red):<br><br>"In fixed-effects negative binomial regression analyses, adjusted for matched-set factors (age, sex, practice, and index date) and smoking status, cases had almost twice the rate of primary care consultations compared with controls (IRR = 1.96, 95% CI 1.94–1.98)."	p. 17<br>Lines:16-18
11. Table 3, 4, 5: While it is in the text, for readers who skip to the table, I suggest adding the model type, and covariates.	We thank you for this comment. We have updated the table note in Tables 3, 4, 5, and 6 to include the model type and covariates:<br><br><br>Table 3:<br>"*Note.* Incidence rate ratios and 95% confidence intervals were estimated using fixed-effects negative binomial regression models. Models compared individuals within matched case-control sets and therefore controlled for age, sex, GP practice, and index date through matched-set fixed effects. Models were additionally adjusted for smoking status. Non-ED controls were used as the reference group. AN = Anorexia Nervosa; ARFID = Avoidant/Restrictive Food Intake Disorder; BED = Binge Eating Disorder; BN = Bulimia Nervosa; CI = Confidence Interval; ED = Eating Disorder; ED-unspecified =	 <br><br><br><br>Table 3, p. 18<br>Lines: 5-12

	unspecified eating disorder; GP = General Practitioner; IRR = Incidence Rate Ratio; OSFED = Other Specified Feeding and Eating Disorder."<br><br>Table 4:<br>"*Note.* Incidence rate ratios and 95% confidence intervals were estimated using fixed-effects negative binomial regression models. Models compared individuals within matched case-control sets and therefore controlled for age, sex, GP practice, and index date through matched-set fixed effects. Models were additionally adjusted for smoking status. White ethnicity and never smoker were used as the reference categories. CI = Confidence Interval; GP = General Practitioner; IRR = Incidence Rate Ratio."<br><br>Table 5:<br>"*Note.* Incidence rate ratios and 95% confidence intervals were estimated using fixed-effects negative binomial regression models including an interaction between ethnicity and ED case status. Models compared individuals within matched case-control sets, therefore controlling for age, sex, GP practice, and index date through matched-set fixed effects. Models were additionally adjusted for smoking status. White ethnicity and non-ED controls were specified as the reference categories. IRRs compare primary care consultation rates between cases and controls within each ethnic group. CI = Confidence Interval; ED = Eating Disorder; GP = General Practitioner; IRR = Incidence Rate Ratio."<br><br>Table 6:	 <br><br><br>Table 4, p. 19<br>Lines: 4-9<br><br><br><br><br><br><br><br><br><br><br><br><br><br><br>Table 5, p. 20<br>Lines: 5-12

	"*Note.* Odds ratios and 95% confidence intervals are from a multivariable logistic regression model with ever receiving an eating disorder referral as the outcome. Cases included in the cohort because of an ED referral, but with no ED diagnosis, were excluded from this analysis. The model includes an interaction between ethnicity and BMI category and adjusts for age, sex, ED diagnosis, IMD quintile, and smoking status. White ethnicity and normal BMI are the reference categories. BMI = Body Mass Index; CI = Confidence Interval; ED = Eating Disorder; IMD = Index of Multiple Deprivation; OR = Odds Ratio."	Table 6<br>p. 22, Lines 1-3, and p. 23 Lines 1-4.
12. "Compared with White individuals, the excess consultation rate among cases was significantly greater for Asian (IRR 1.12)" I could not follow how this would be interpreted as excess consultations, as this was not previously mentioned in the paper - could the authors please clarify?	Thank you for this comment and the opportunity to clarify our results. We intended to describe that, compared with White individuals, the relative increase in consultation rates associated with ED case status was greater among individuals from Asian, Black, Mixed, and Other ethnic backgrounds. We have revised the presentation of these results to make this interpretation clearer (changes in red):<br><br>*"Compared with White individuals, the relative increase in consultation rates between ED cases and controls was significantly greater among Asian (IRR 1.12), Black (IRR 1.24), Mixed (IRR 1.12), and Other ethnic groups (IRR 1.64)."*	p. 19<br>Lines:14-17
Discussion<br><br>13. The study date range of 2010-2023 is quite wide. While it strengthens the statistical power, it may also risk introducing	Thank you for this comment. To control for temporal trends, we used index date as one of our matching criteria and everyone has the same follow up period (24 months). This ensured that both groups were subject to the same clinical	

temporal heterogeneity. It is possible that there are temporal trends such as changes in clinical practice, EHR data quality or recording/coding practices, or changes in awareness of ED which lead to changes in patient behaviour (e.g. more willing to request referrals).<br><br>It would be interesting in the discussion to hear the authors thoughts on this possibility, and whether it impacts the results. It could also be informative to describe the distribution of cases, e.g. a histogram of index dates.	practices and coding standards at any given time point.<br><br>We added the following to the Methods section to make this more clear:<br><br>*"This ensured that the controls were under clinical observation during the same time as their matched case, reducing the likelihood of selecting controls with infrequent primary care use. It also helped to account for temporal changes in clinical practice, coding behaviour, and EHR recording over the study period."*<br><br>And this exists alongside this existing information in the Statistical Analyses section of the manuscript:<br><br>"All outcomes were ascertained within a fixed 24-month pre-index window for every participant (24 months prior to diagnosis/referral). As follow-up time was identical for all individuals, no offset for person-time was required and model estimates compare consultation counts over equivalent 24-month observation windows."<br><br>We have also added text to the Discussion acknowledging that temporal changes in diagnostic frameworks across the study period may have influenced who was eligible for diagnosis over time:<br><br>*"In addition, the study spans several diagnostic classification frameworks for EDs including DSM-IV, DSM-5, ICD-10 and ICD-11. The introduction of the DSM-5 and ICD-11 broadened ED diagnostic criteria and may have*	 <br><br>p. 8<br>Lines: 22-25<br><br><br><br><br><br><br><br><br><br><br>p. 13<br>Lines: 25-27 and p. 14 Lines: 1-2.<br><br><br><br><br><br><br><br><br><br><br><br>p. 27, Lines 3-7

	increased the number of individuals eligible for diagnosis over time, though this is unlikely to substantially affect the study's primary focus examining demographic differences in health care use."	
**Reviewer #2 Dr. Paul Jenkins, University of Reading**		
Thank you for the opportunity to review this paper, which looks at frequency of contact in primary care by adults with a diagnosis of an ED. The manuscript is generally clear, presentation of data (including in SM) seems thorough, and the findings are important. I have some comments the authors might wish to consider.	Thank you for this positive feedback.	N/A
I think the use of "UK" is potentially misleading (given that data from Wales and Scotland are not included), and I invite the authors to think about this, mindful that this could become complicated. Population figures might also need to be adjusted should this language be changed.	We thank the reviewer for this feedback. At your suggestion, we have revised the title of the manuscript to be clearer that the focus was on English practices.<br><br>Revised title: "Primary care use among adults with eating disorders in England: a population-based cohort study using electronic health records"	p. 1, Lines 2-3
Why was an upper limit of 65y used? Similarly, the timeframe of 24m could be better justified. Is it possible to say whether there are patterns within this 24m – e.g., clustering at the beginning, or end?	Thank you for the opportunity to clarify the rationale for our study design. We restricted the cohort to adults aged 18-65 years to focus on a working-age adult population and reduce the influence of age-related multimorbidity and health care use patterns that become increasingly common in older adults.[1] Previous studies examining EDs and serious mental illness in CPRD have similarly applied upper age limits around 60 years.[2,3] We have added the	

	following clarification of this rationale to the Methods section:<br><br>*"Participants were restricted to adults aged 18–65 years, consistent with comparable CPRD studies examining EDs and mental health populations, to facilitate comparison with existing literature (21, 22)."*<br><br>1. Cassell, A., Edwards, D., Harshfield, A., Rhodes, K., Brimicombe, J., Payne, R., & Griffin, S. (2018). The epidemiology of multimorbidity in primary care: a retrospective cohort study. British Journal of General Practice.<br>2. Kontopantelis, E., Olier, I., Planner, C., Reeves, D., Ashcroft, D. M., Gask, L., ... & Reilly, S. (2015). Primary care consultation rates among people with and without severe mental illness: a UK cohort study using the Clinical Practice Research Datalink. BMJ open, 5(12), e008650.<br>3. Jeffery, A., Hayes, J. F., Launders, N., Lewis, G., Osborn, D., Bould, H., ... & Solmi, F. (2025). Mortality and hospital admissions in people with eating disorders: longitudinal cohort study in secondary care-linked English primary care records. The British Journal of Psychiatry, 1-9.<br><br>We selected a 24 month timeframe because evidence suggests EDs are frequently associated with prolonged durations of untreated illness prior to diagnosis or treatment, often extending to up to two years for AN or six years for BED.[4] A 24-month pre-index window was chosen pragmatically to balance adequate pre-diagnosis observation while avoiding overly	p. 8<br>Lines: 5-7

	restrictive requirements for long periods of continuous GP registration. We have clarified this rationale in the manuscript:<br><br>*"Healthcare use was observed over a 24-month period. This window was chosen to capture the prolonged period of untreated illness commonly observed prior to ED diagnosis and referral (23)."*<br><br>4. Austin, A., Flynn, M., Richards, K., Hodsoll, J., Duarte, T. A., Robinson, P., ... & Schmidt, U. (2021). Duration of untreated eating disorder and relationship to outcomes: A systematic review of the literature. European Eating Disorders Review, 29(3), 329-345.	p. 8<br>Lines: 7-9
I was a little unclear on how the codelist was used (mindful that variations have been used before). For instance, if "anorexia" was noted, how was this distinguished from "anorexia nervosa"? A little more detail is important for readers to have confidence that the psychiatric conditions of EDs are being considered, rather than 'organic' or non-specific issues (e.g., loss of appetite).	Thank you for your comment and the opportunity to clarify this part of our methodology. We included the terms 'appetite loss - anorexia' and 'anorexia' as in UK primary care, GPs frequently use 'anorexia' as a pragmatic clinical shorthand for Anorexia Nervosa (AN) during time-pressured consultations. Restricting the code list strictly to 'Anorexia Nervosa' would likely lead to significant under-identification, missing cases where the clinician recorded the primary diagnostic term available in the search dropdown. We have clarified how codelists were developed in the text:<br><br>*"We used previously published codelists alongside novel codelists developed for this study, following published guidance for routinely collected electronic health data. All codelists were reviewed by*	p. 9<br>Lines:3-6

	clinicians with expertise in primary care and EDs (24).”<br><br>And in Table 1:<br><br>*“Previously validated ED codelists were updated for the present study to identify Read and/or SNOMED CT medical codes corresponding to categories from DSM-IV, DSM-5 and ICD-10. Only codes representing clinical ED diagnoses were included; non-specific symptom codes (e.g., loss of appetite) were excluded. The terms ‘appetite loss - anorexia’ and ‘anorexia’ were retained, as excluding them may have missed records where this shorthand was used for AN in primary care coding.”*	Table 1, p. 10
What was the cutoff (e.g., in mmol/L) for hypokalaemia used? (I think 3.5 is mentioned in the SM, which could be stated in the main text.) Given that this did not really feature in the analyses, some of the detail (e.g., in Table 1) might be removed, perhaps only keeping reference to the fact that this was planned as a covariate, but not used (p. 13).	Thank you for this comment. A cutoff of <3.5 mmol/L was used to define hypokalaemia, consistent with standard clinical definitions.<br><br>We agree that because it does not feature in our analyses we removed it from Table 1, only keeping reference to the fact it was planned (and why) in our description of Statistical Analyses (changes in red):<br><br>*“We had originally planned to include hypokalaemia as a covariate as a proxy indicator of ED severity (SM 2.3), given its association with recurrent purging behaviors (e.g. vomiting, laxative, and diuretic abuse) and its occurrence in severe ED presentations (32). However, only a small proportion of participants met criteria for hypokalaemia (6.97%), and the variable was therefore excluded from the final analytical models.”*	p. 14 Lines: 25-28 and p. 15 Lines: 1-2.

Why were analyses adjusted for smoking status (only)?	Thank you for this comment. Smoking status was included as a covariate because it is associated with both physical and mental health conditions and patterns of healthcare use, including primary care consultation frequency.[1] Smoking is also routinely recorded in UK primary care databases and commonly used as an adjustment variable in CPRD-based epidemiological research.<br><br>We have added the following clarification into the manuscript:<br><br>*"Smoking status was included as a covariate because it was not part of the matching strategy and may be associated with healthcare use (30)."*<br><br>1. Atkinson MD, Kennedy JI, John A, Lewis KE, Lyons RA, Brophy ST; DEMISTIFY Research Group. Development of an algorithm for determining smoking status and behaviour over the life course from UK electronic primary care records. BMC Med Inform Decis Mak. 2017 Jan 5;17(1):2. doi: 10.1186/s12911-016-0400-6.<br><br>We additionally controlled for several variables through the matched case–control design itself. Specifically, fixed-effects models compared individuals within matched sets and therefore adjusted for age, sex, GP practice, and index date. Other covariates were included depending on the specific research question. For example, BMI was examined in sensitivity analyses because we considered it may act as a mediator rather than a confounder in the	 <br><br><br><br><br><br><br>p. 13<br>Lines:16-17

	relationship between ED diagnosis and healthcare utilisation. Variables such as IMD quintile and ED diagnosis subtype were included in models examining referral outcomes.<br><br>We have revised our Methods section to more clearly describe how we adjusted for covariates in our analyses:<br><br>*"Models compared individuals within matched case-control sets. Age, sex, GP practice and index date were therefore controlled by design through matching and by inclusion of matched-set fixed effects. Smoking status was included as a covariate because it was not part of the matching strategy and may be associated with healthcare use (30)."*<br><br>*"To address Research Question 3, whether ethnicity predicted a referral to ED specialist services, we fitted a multivariable logistic regression model with ever receiving an ED referral (yes/no) as the outcome. Cases included in the cohort on the basis of an ED referral alone, with no recorded ED diagnosis, were excluded from this analysis because referral was the outcome being modelled. An interaction between ethnicity and BMI category was included to assess potential effect modification, and models adjusted for age, sex, ED diagnosis, IMD quintile, and smoking status."*<br><br>And in each of the Table notes (please see Reviewer #1 comment 11).	p. 13<br>Lines:13-17<br><br>p. 14<br>Lines:3-9
A potential limitation which could be noted (e.g., p. 26) is that language proficiency (for	Thank you for this suggestion. We have integrated your comment into the limitations section of our discussion:	

minoritised groups, but perhaps more widely) was not considered / available, and this could be related to uptake / access.	*"Additionally, cultural and linguistic differences in how symptoms are described and communicated during healthcare encounters may also influence recognition, clinical diagnosis, and referral decisions across ethnic groups (Robinson et al., 2021)."*	p. 27<br>Lines: 19-22
Statistics 'demonstrating' equivalence between the two groups might be useful in Table 2.	We thank you for this comment. We have now assessed baseline comparability between cases and controls using standardized mean differences (SMDs), which are less sensitive to sample size than significance testing.<br>As expected, given our matched design, age and sex were well balanced (SMDs<0.02), and IMD showed minimal difference (SMD=0.08). Some differences were observed for ethnicity (SMD = 0.203) and BMI (SMD =0.420). Differences in BMI would be expected clinically in an ED population and differences in ethnicity are consistent with previous research that individuals from minoritised ethnic backgrounds are less likely to be diagnosed with EDs[1]. These variables were included as covariates in the regression models.<br><br>1. Sonneville, K. R., & Lipson, S. K. (2018). Disparities in eating disorder diagnosis and treatment according to weight status, race/ethnicity, socioeconomic background, and sex among college students. International Journal of Eating Disorders, 51(6), 518-526.<br><br>The SMDs have been added to Table 2.	Table 2, p. 15-16

A citation is needed (p. 23, ll26–27) regarding "evidence that Black individuals… often have higher BMI and more severe eating-related concerns" than community samples.	Thank you for this comment. We have now added an appropriate citation for this statement:<br><br>"This is interesting in the context of existing evidence that Black individuals presenting for treatment of EDs often have higher BMI and more severe eating-related concerns compared with community counterparts (46)."<br><br>Citation: Grilo CM, Lozano C, Masheb RM. Ethnicity and sampling bias in binge eating disorder: Black women who seek treatment have different characteristics than those who do not. Int J Eat Disord. 2005;38(3):257-62	p. 26, Line 5
The findings on referral rates are interesting (albeit disappointing). Are the authors able to comment on whether this categorisation covers 'attempted' referrals, and/or whether these were 'accepted' by the EDS? This might then affect GP contact rates, although perhaps not in predictable directions (e.g., referral to EDS reduces GP contact as patient is under a 'specialist' but could also increase contact due to requests for medical monitoring).	We thank the reviewer for this comment. Our referral variable captured referrals recorded within primary care records, but we were unable to determine whether these referrals were accepted and resulted in treatment at specialist ED services. As this study did not include linked secondary care data (e.g. HES secondary care records), we could not confirm whether referrals resulted in assessment or treatment within specialist services. We have clarified this limitation in the revised manuscript and highlighted it as an important area for future research:<br><br>In the Limitations:<br>*"Our study did not include access to linked secondary care data (e.g. Hospital Episode Statistics or Mental Health Services Data Set) and so we were also unable to determine whether referral events logged in primary care were accepted by specialist ED services or resulted in treatment. This may be*	 <br><br><br><br><br><br><br><br><br><br><br><br><br><br><br>p. 27<br>Lines: 23-27 and p. 28 Lines: 1-2.

	particularly important as in the UK, one in seven NHS patients referred from primary to secondary care services fall into a 'referral black hole', where referrals are delayed or rejected, highlighting potential gaps in care even after the referral has been initiated (Healthwatch England, 2026)."<br><br>In the 'Unanswered questions and future research' section:<br><br>*"Furthermore, linking primary care records with secondary care hospital episode statistics would move beyond patterns of presentation to examine outcomes following referral, specifically whether referrals result in specialist assessment and treatment in secondary care."*	p. 29<br>Lines: 11-13
	We also agree that it would be useful to examine primary care use following referral to specialist treatment, however, it is beyond the scope of the present study. We have therefore made our recommendation for this as an area of future research more clear in the Future Research section of the revised manuscript (change in red):<br><br>*"The present study focused on the two year period prior to a diagnosis or referral and it would be useful to examine whether these patterns persist or change in primary care in the period after diagnosis and/or treatment (5)."*	p. 29<br>Lines: 16-19

There are two findings which stood out to me and which I felt were discussed in limited depth. First, the 'prevalence' of AN was notably high relative to other EDs, particularly in light of community estimates (e.g., van Eeden et al., 2021, Current Opinion in Psychiatry). Could the authors comment on why the number of such cases was so apparently high in the current study?	Thank you for your comment. We have added the following to our discussion:<br><br>*"Furthermore, a relatively high proportion of individuals in our cohort were diagnosed with AN (43%), which may reflect both the medical risk associated with AN and its clinical visibility, with individuals more likely to present earlier to healthcare services and require hospitalisation compared with other ED presentations (46)."*	p. 25<br>Lines: 25-28
Relatedly, the OSFED category showed fairly high primary care use and other idiosyncrasies (e.g., wide BMI range). Could the authors comment on the types of presentations which might fall into this group, even if that is broad? Given its relative prevalence (as the third most common diagnostic label) and issues around identification (e.g., Attia & Guarda, 2024, IJEDs; Jenkins et al., 2021, BJPsych Open), further comment on this might be warranted.	We thank you for your comment and the opportunity to strengthen our interpretation of the findings. We have added the following to the Discussion section of our revised manuscript:<br><br>*"Although OSFED has been reported to represent a substantial proportion of ED presentations in community and clinical populations (43), relatively few individuals in our cohort received a specific OSFED diagnosis (6.6%). This may partly reflect the study observation period spanning both DSM-IV and DSM-5 diagnostic classifications, with OSFED only introduced following publication of DSM-5 (44). Instead, a large proportion of participants either had no recorded ED diagnosis despite referral to specialist services or had a non-specific "Eating Disorder" diagnostic code. This pattern is consistent with previous CPRD-based ED studies and may reflect challenges distinguishing between ED presentations in routine primary care settings (1). Notably, individuals with OSFED and ED-unspecified diagnoses demonstrated higher primary care consultation rates to those with AN and*	p. 25<br>Lines: 13-24

	BN, potentially reflecting challenges identifying ED presentations outside of AN and BN or differences in how these presentations are managed in primary care (45).”	
Finally, this might be outside of the scope of the research, but I was curious whether contact rates varied according to ED severity as specified in the DSM, for instance. It may not be possible (e.g., p. 24, ll41–43), but it would be interesting if these data were available.	Thank you for this suggestion. We agree that examining whether primary care consultation rates vary according to ED severity would be clinically informative. However, ED severity is not routinely coded within CPRD Aurum, and therefore could not be directly derived for the present study. While proxy indicators of severity (for example, medical complications such as hypokalaemia or other comorbidities) could potentially be explored, we considered this beyond the scope of the current analysis. We have now acknowledged this as an important area for future research in the Discussion section.<br><br>*“It could also be informative to explore whether consultation patterns differ according to ED severity, although severity is not routinely coded within CPRD Aurum and may require the development of proxy indicators using clinical comorbidities.”*	p. 29 Lines: 8-11
Some minor suggestions:<br><br>There is some repetition (of primary care) – e.g., in the Title, objectives<br><br>The Title could specify that this was a study of adults	We thank you for your comments. We have aimed to reduce the repetition of ‘primary care’ in our title and objectives and also revised our title to indicate that this is a study with adults.<br><br>New title: *“Primary care use among adults with eating disorders in England: a population-based cohort study using electronic health records”*	p. 1, Lines 2-3<br>p. 2, Lines 2-3

There are some typographical errors (e.g., Abstract – "had diagnosis of anorexia"; "detailed by Wyatt et al.'s (26) to identify"; "studies [e.g. (26-28) and"; "These finding")<br><br>HER should be EHR (p. 7)<br><br>Pica does not need to be capitalised (several places)	Thank you for highlighting these typographical errors. We have carefully reviewed the manuscript for errors and made corrections throughout.	Throughout the manuscript.
Some phrasing could be more specific, e.g., participants were "required to be ED free" – presumably this refers to an absence of an ED diagnosis on their records?	We thank you for your comment. We have clarified how controls were defined in the Methods section (changes in red):<br><br>"Controls were required to have no recorded ED diagnosis or referral in their CPRD record, based on the study codelists (Table 1)."	p. 8<br>Lines:18-20
Individuals with EDs are noted as having "higher comorbidities" – relative to whom? Non-ED cases? Other MH problems?	Thank you for your comment. We have clarified this statement in the manuscript (change in red):<br><br>"This is consistent with evidence that individuals with ED have higher comorbidities of both physical and mental health problems compared with individuals without EDs which is likely to impact primary care use (1, 3)."	p. 24<br>Lines:15-17

VERSION 2 - REVIEW

Reviewer 2

Name Jenkins, Paul

Affiliation University of Reading, School of Psychology and Clinical Language Sciences

Date 30-May-2026

COI

Many thanks for your attention to my previous comments and suggestions. I believe the paper is much clearer now. I only have some minor remaining comments:

1. "etc" is not needed after "e.g." (p. 12)

2. As the Title has changed, the limitation that the study does "not include the primary care use of individuals from Scotland or Wales" (p. 27) is moot. That said, it could be reframed to suggest that a UK-wide study might be useful.

3. Whilst I agree with the authors that "anorexia" may commonly be used as a shorthand for AN (although I'm not sure I would describe it as "pragmatic"!), it seems there remains a risk that some patients in this study could be considered as a "case" (of AN) when they have no ED (psycho)pathology. If this interpretation is correct, this should be noted as a limitation.

VERSION 2 - AUTHOR RESPONSE

Reviewer: 2
Dr. Paul Jenkins, University of Reading
Comments to the Author:
Many thanks for your attention to my previous comments and suggestions. I believe the paper is much clearer now. I only have some minor remaining comments:

1. "etc" is not needed after "e.g." (p. 12)

We thank the reviewer for noting this typographical error. We have removed it from the manuscript so the sentence now reads:

Lines 232-234:

"Using consultation source codes, we first excluded codes that were administrative in nature (e.g. "report", "document") or clearly outside the scope of primary care (e.g. "Accident and Emergency")."

2. As the Title has changed, the limitation that the study does "not include the primary care use of individuals from Scotland or Wales" (p. 27) is moot. That said, it could be reframed to suggest that a UK-wide study might be useful.

We thank the reviewer for this comment. We have removed the limitation that the study does not include primary care use for individuals from Scotland and Wales on p. 27. And have added the following to the *unanswered question and future research* sub-section of the Discussion.

Lines 601-603:
"As analyses were restricted to data from England and a small number of practices in

Northern Ireland, future studies incorporating GP practices across all UK nations would

help assess the generalisability of these findings.”

3. Whilst I agree with the authors that "anorexia" may commonly be used as a shorthand for AN (although I’m not sure I would describe it as "pragmatic"!), it seems there remains a risk that some patients in this study could be considered as a "case" (of AN) when they have no ED (psycho)pathology. If this interpretation is correct, this should be noted as a limitation.

Thank you for this comment. We have expanded the discussion to more clearly describe how differences in clinician coding practices may have affected the accuracy of recorded ED diagnoses and to acknowledge this as a potential limitation of the study:

Lines 522-529 (changes in red):

“The current study is limited by differences in coding practices for individual clinicians (GPs), which may impact the consistency and accuracy of ED diagnoses and consultation events. For example, ‘anorexia’ was retained in the diagnostic codelist to avoid missing cases where it was used as a shorthand for AN, however, the term may also be used more generally to indicate loss of appetite. Inclusion of this code may have resulted in some individuals being classified as having AN despite not having an ED diagnosis. The implication of this potential misclassification is that our results may underestimate the true differences between cases and controls.”
